# The homogenous alternative to biomineralization: Zn- and Mn-rich materials enable sharp organismal “tools” that reduce force requirements

**DOI:** 10.1038/s41598-021-91795-y

**Published:** 2021-09-01

**Authors:** R. M. S. Schofield, J. Bailey, J. J. Coon, A. Devaraj, R. W. Garrett, M. S. Goggans, M. G. Hebner, B. S. Lee, D. Lee, N. Lovern, S. Ober-Singleton, N. Saephan, V. R. Seagal, D. M. Silver, H. E. Som, J. Twitchell, X. Wang, J. S. Zima, M. H. Nesson

**Affiliations:** 1 Department of Physics, University of Oregon, 1274, Eugene, OR 97403 USA; 2 Physical and Computational Sciences Directorate, Pacific Northwest National Laboratory, Richland, WA 99354 USA; 3 Department of Biochemistry and Biophysics, Oregon State University, Corvallis, OR 97331 USA

**Keywords:** Biomaterials, Biophysics, Ecology, Physiology

## Abstract

We measured hardness, modulus of elasticity, and, for the first time, loss tangent, energy of fracture, abrasion resistance, and impact resistance of zinc- and manganese-enriched materials from fangs, stings and other “tools” of an ant, spider, scorpion and nereid worm. The mechanical properties of the Zn- and Mn-materials tended to cluster together between plain and biomineralized “tool” materials, with the hardness reaching, and most abrasion resistance values exceeding, those of calcified salmon teeth and crab claws. Atom probe tomography indicated that Zn was distributed homogeneously on a nanometer scale and likely bound as individual atoms to more than ¼ of the protein residues in ant mandibular teeth. This homogeneity appears to enable sharper, more precisely sculpted “tools” than materials with biomineral inclusions do, and also eliminates interfaces with the inclusions that could be susceptible to fracture. Based on contact mechanics and simplified models, we hypothesize that, relative to plain materials, the higher elastic modulus, hardness and abrasion resistance minimize temporary or permanent tool blunting, resulting in a roughly 2/3 reduction in the force, energy, and muscle mass required to initiate puncture of stiff materials, and even greater force reductions when the cumulative effects of abrasion are considered. We suggest that the sharpness-related force reductions lead to significant energy savings, and can also enable organisms, especially smaller ones, to puncture, cut, and grasp objects that would not be accessible with plain or biomineralized “tools”.

## Introduction

There are two distinct classes of inorganic-enriched biological materials used in claws, teeth, stings and other animal “tools”. The first class, biomineralized tissues, employ mainly Ca-, Fe-, and Si-based minerals, like those in calcified teeth and bones or in the magnetite-filled teeth of certain mollusks. These biomineralized tissues contain two separate phases: an organic matrix and mineral inclusions that are usually larger than 100 nm in scale. We term the second class of enriched materials ‘Metal-Halogen’ or ‘Heavy Element Biomaterials’ (HEBs here) because the dominant inorganic elements are heavy metals or heavy halogens—zinc, manganese, bromine, and copper, in concentrations between about 1 and 25% of dry mass. While not as well understood as biomineralization, the HEBs are at least as widely employed, occurring in many insect orders, in spiders and most other arachnids, in many centipedes, crustaceans, marine worms and members of other phyla^1–22^. The HEBs are distinguished from biomineralized tissue by the apparent lack of separate organic and inorganic phases^14^. Here, we investigate potential adaptive advantages associated with HEBs by measuring a range of mechanical properties, and we investigate their composition on a nanometer scale.

### Mechanical properties

**Figure 1:**
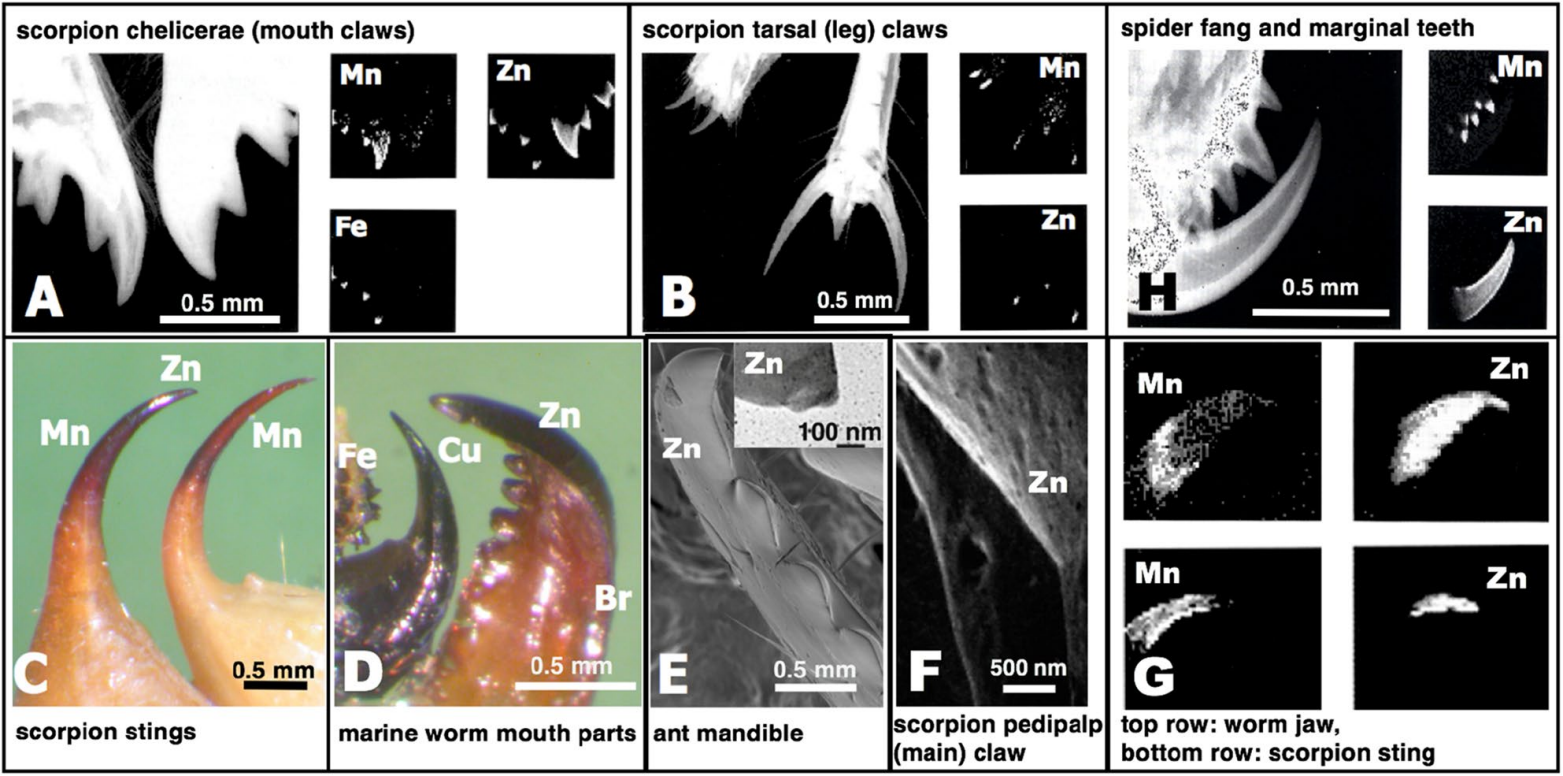
Images of HEB-containing “tools” with the dominant inorganic element indicated. (**A**) Portions of the chelicerae (mouth claws) of the scorpions *Centruroides exilicauda* (left) and *Paruroctonus mesaensis* (right). These maps, and those in (**B**), (**G**) and (**H**), show projected mass (large image) and element (small images) density, and were made using high-energy ion microscopy, which differs from electron microscopy in that it samples the entire volume of these specimens instead of a surface layer^12^. (**B**) Leg claws of the scorpions *Centruroides exilicauda* (left) and *Vaejovis confusus* (right). (**C**) Stings of the scorpions *Vaejovis confusus* (left) and *Centruroides exilicauda* (right). (**D**) “Tools” of marine worms: paragnaths of *Nereis vexillosa* (left), jaw of *Glycera convoluta* (middle), jaw of *Nereis vexillosa* (right). (**E**) SEM image of the mandible of the ant *Atta cephalotes*, showing the cutting edge, measured to have a 50 nm edge radius (TEM image of cross-section in inset). (**F**) SEM image of sharp ridges on a pedipalp tooth of the scorpion *Paruroctonus boreus*. (**G**) Similar pattern of Zinc in the tips and manganese in the shafts in “tools” from two different phyla. Top row: jaws of the worm *Nereis virens*, bottom, sting of the scorpion *Vaejovis confusus*. (**H**) Chelicera of the spider *Araneus diadematus,* showing the large fang and the marginal teeth. The high aspect ratios (length/width) minimize the force required for depth penetrated^23^.

While biomineralized tissues are often filled with minerals to a degree that the mechanical properties are clearly altered, it is not as obvious that the mechanical properties of HEBs are distinct from properties of similar biomaterials that are not enriched (referred to here as non-HEBs). However, the location of HEBs in the contact regions of “tools” such as teeth, claws, jaws and stings (Fig. 1) motivates the investigation of mechanical properties.

Only hardness and, in a few cases, modulus of elasticity of Zn- and a few Mn-HEBs have previously been measured^3,7,9,10,14–16,24–29^. Most results have indicated that Zn-HEBs are harder than surrounding regions, although most of these measurements were made on dry or re-wetted sections instead of fresh contact surfaces, and the relative hardness of HEBs and non-HEBs can change with drying^30^.

The role of zinc in hardening the Zn-HEBs has been supported by the observation that ant teeth harden relative to surrounding cuticle in the short period of time that zinc is incorporated late in cuticle development^31,32^, and by the observation that hardness drops when Zn is removed from worm jaws and increases again as it is replaced by zinc, manganese, or copper^33,34^.

In addition to hardness and modulus of elasticity, abrasion resistance is of particular interest for many biological “tools” because they cannot be re-sharpened, and this interest has led to predictions and claims of outstanding HEB abrasion resistance from models that used measurements of hardness and modulus of elasticity to predict abrasion resistance^35–37^. But, these models did not correctly predict even the ranking of abrasion resistance in direct abrasion tests of bromine-enriched cuticle in the claw tips of certain crab species and comparison materials, highlighting the need for direct testing^14^.

Along with the direct abrasion resistance tests on crab claws, we also measured energy of fracture and damping properties, which had not previously been reported for HEBs. While the tips of these crab claws contained bromine, the rest of the claws, like those of most crabs, were highly calcified. This crab cuticle presented a unique opportunity to compare the advantages of biomineralized and HEB materials in a single organism and “tool”.

The calcified cuticle had a 3-times higher hardness value and a nearly 5-times higher modulus of elasticity than the Br-HEB, but the calcified cuticle was much more susceptible to fracture, requiring about 1/9th as much energy to produce the same area of fracture. The advantage of the Br-HEB appeared to be that it was moderately (roughly 1.5 times) harder and stiffer than HEB-free arthropod cuticle, but did not increase susceptibility to fracture. An additional fracture-related advantage of the Br-HEB was that it was a highly-damped material (about 6-times higher loss tangent than the calcified cuticle), which would lead to more rapid absorption of energy from impacts than calcified cuticle.

An important difference between the biomineralized and Br-HEB regions of the claws was that the largest structures observed in the Br-HEB were 7 nm laminae, about 1/1000 of the size of mineral inclusions in the calcified cuticle.

The crab claw measurements suggested that calcified cuticle would be best for regions designed to apply large pressures, but that were not sensitive to fracture, and that the brominated cuticle would be optimal for sharp edges and tips that must keep their shape under pressure and not fracture. This was consistent with the observed crab behavior. The HEB regions were used as forceps while the calcified regions were used for crushing, and were often found to be chipped.

In this paper, we extend the suite of measurements made on crab cuticle to smaller samples, Mn- and Zn-HEBs and surrounding regions, in a species of nereid worm, spider, ant and scorpion. In addition to hardness and modulus of elasticity, we measured values of properties that had not previously been reported for any Mn- or Zn-HEBs, namely, loss tangent, energy of fracture, abrasion resistance and impact resistance. We also include some measurements on biomineralized and unmodified organic “tools” for comparison. And we include measurements of standard engineering materials made using the same techniques because properties of HEBs must necessarily be measured on small scales (e.g. the scale of ant mandibular teeth), and materials may have scale-dependent properties^14^.

Mechanical properties such as hardness and fracture resistance may be particularly biologically relevant for small organisms that overcome force limitations with sharp cutting and puncturing “tools”^23^. Fracture and other damage to “tools” is more likely to be fatal for smaller organisms that cannot provide the increased forces necessary when using blunted tools. Younger organisms that are smaller and more force limited may be even more reliant on sharp “tools” than adults. For eusocial organisms, damage and wear may not be fatal, but may require changes in behavior. For example, leafcutter ants with highly worn mandibles discontinue cutting but will carry leaf fragments back to the nest^38^. This behavioral change illustrates the importance of having sharp “tools”, as do measurements that show that ants cutting outside the nest with the least worn mandibles cut about twice as fast as ants with average levels of wear^38^.

The role of food fracture, tool wear, tool shape and their ecological implications have been explored for many trophic interactions ^39–44^. Here we introduce estimates of the advantages of different materials in “tools”, based on measurements of their material properties. We attempt to quantify the advantages of different materials with estimates of differences in required force, energy and muscle mass.

### Composition

It is difficult to account for the 10% or 20% concentrations of zinc in Zn-HEBs, except through biomineralization. But zinc-rich regions in ant and scorpion “tools” did not appear to contain biominerals in X-ray or electron diffraction experiments, nor was there evidence of electron-dense inclusions at a TEM resolution of about 5 nm^32^. Several subsequent investigations have also not found evidence of biominerals in Zn-HEBs^15,36^.

In addition, no evidence of biomineralization was found in Extended X-ray Analysis of Fine Structure (EXAFS), which was used to examine worm jaws, ant mandibles, and spider fangs from the species studied here, as well as from the forcipule of a centipede and cheliceral tynes of a vaejovid scorpion^13^. For each of the samples examined, EXAFS indicated that zinc is 4-coordinate and bound to O and/or N (~ 2 Angstrom peaks). But, no Zn–Zn scattering signal was found, though such a signal would be expected even for amorphous biominerals. These results suggest that either the zinc-atoms are further apart than about 0.6 nm, or else that the Zn–Zn distance is highly variable. Zn-HEB EXAFS spectra were similar to spectra for zinc in concentrated aqueous hydroxide^45^, possibly because of highly variable Zn–Zn distances.

The earliest suggested binding mechanism, made before the magnitudes of the zinc concentrations were clear, was as individual zinc atoms cross-linking organic molecules in the cuticle^3^. Amino acid analysis and sequencing of the dominant protein from the tips of zinc-enriched worm jaws, indicated that the fraction of histidine residues could be as high as about 27%, which has been interpreted as supporting a hypothesis that zinc cross-links proteins as Zn(His)_4_ or Zn(His)_3_Cl^27,33,34,46^. However, if all of the zinc were bound as Zn(His)_3_Cl, the maximum possible concentration would be about 5% zinc. In contrast, we have measured concentrations of as high as 18% in nereid worms^7^. The difficulty of orienting virtually every histidine residue in every protein molecule so that each zinc atom can bind to 3 or 4 histidines also suggests that binding to multiple histidines is unlikely to be the dominant binding mechanism.

EXAFS data also indicated that only a minor fraction of the zinc could be bound to 3 or 4 histidine residues. The EXAFS “outer shell” structure differed between organisms but was consistent in all cases with some binding to imidazoles, as were more recent data from XANES and EELS for spider fangs^47^. However, modeling suggested that the low level of “outer shell” structure for arthropods^13^ was consistent with only about one in four of the binding sites being on an imidazole, while, for the nereid worm jaws, two imidazoles were possible. One possibility that is consistent with all observations is that zinc is mainly bound to a single histidine residue and to water as a hydroxide.

The specialized zinc binding surface layer in arthropods is contiguous with and thus thought to be a modified layer of the exocuticle^32^. This modified layer appears to be dominated by proteins and zinc, with little or no chitin. No evidence of chitin crystallinity was found in the outer layer^27^ and we recently confirmed this for leafcutter ants, finding that colloidal gold-labeled wheat germ agglutinin (WGA), which binds specifically to N-acetyl glucosamine, binds in much lesser quantities in the zinc-region than in surrounding regions of cuticle.

We have also used EXAFS to examine the Br-HEB located at the tips of certain crab claws, finding that the Br is most likely bound in singly-brominated phenyl rings^14^.

This binding motif appears to be widespread: the Br EXAFS spectra for claw spoon tips of the crab *P. crassipes* were similar to spectra from the tarsal claw tips of another shore crab species, and similar to the spectra from Br-enriched mechanical structures of several other invertebrate phyla: nereid polychaetes, a pycnogonid, and a priapulid^14^. EXAFS of the Br in the nereid jaws was consistent with singly brominated tyrosine, but not multiply-halogenated tyrosines observed elsewhere^46^.

The generality of the chemical environment of zinc and bromine, across multiple phyla, is a main justification for grouping the many examples together as zinc- or bromine-HEBs.

Here we use Atom Probe Tomography to further examine the binding and spatial distribution of zinc.

## Methods

Because biological materials are often viscoelastic composites, with properties dependent on orientation as well as spatial and temporal scales, all tests were designed to mimic natural conditions of tool use. For example, because of possible anisotropies, indentations were made on contact surfaces instead of cross sections of the “tools”; abrasion resistance was measured in the contact direction. Fracture tests were designed to mimic the tension on the side of a tooth or sting subjected to lateral forces; the velocities in our impact tests were between 0.1 and 1 m/s, typical for tool interactions, though 1/10 as fast as for some organisms^48,49^.

### Organisms

All organisms, except the salmon, were housed alive in our laboratory until just before testing. Samples were measured within 24 h of removal from the organism, and were maintained in high-humidity environments during the preparation period. This is particularly important because mechanical properties can depend strongly on water content and this dependence can differ between materials. For example, non-HEBs can harden more than HEBs with drying^30,50,51^.

1. Leafcutter ants, *Atta cephalotes,* were obtained from colonies we collected in Flores, Guatemala and Arena Forest Reserve, Trinidad. They were maintained on Himalayan blackberry leaves, *Rubus armeniacus*, Portugese laurel, *Prunus lusitanica*, and Japanese spurge, *Pachysandra terminalis*.
2. Nereid worms, *Neanthes brandti* (synonymous with *Alitta brandti*), were collected from trenches dug in bars near the mouth of the Coos River, Charleston, Oregon. They were kept in sea-water in a laboratory refrigerator at about 5 °C.
3. Scorpions, *Hadrurus arizonensis,* from Arizona, were obtained from commercial suppliers (e.g. Bugs of America, http://bugsofamerica.com) and fed House crickets, *Acheta domesticus*, and mealworms, *Tenebrio molitor* larvae*.*
4. Spiders, *Araneus diadematus,* were collected seasonally around the University of Oregon campus, and kept alive in a laboratory refrigerator at about 5 °C. Tarantulas, *Aphonopelma hentzi* were obtained from Carolina (https://www.carolina.com).
5. Chitons, *Katharina tunicata*, and *Cryptochiton stelleri* were collected in rocky intertidal regions along the coast near Charleston, Oregon and kept alive in a laboratory refrigerator.
6. Salmon heads, *Oncorhynchus* sp., were obtained fresh from seafood stores.
7. Leaf cutter bees, *Megachile rotundata,* were obtained from Mason Bees for Sale (www.masonbeesforsale.com).

### Hardness, modulus of elasticity and damping measurements

Hardness, modulus of elasticity and dynamic mechanical property measurements were made by pressing a sharp diamond probe into specimens and measuring the resulting indentation as it changed in time. A higher modulus of elasticity indicates that a structure is stiffer and suffers less elastic (quickly recovered) deformation. A higher hardness value indicates that the material will undergo less plastic (non-recovered) deformation and thus will have a smaller pit left behind after the indentation. A material with a higher loss tangent will absorb more energy of vibration (higher damping), and is characterized by lagging surface deformation and recovery (viscoelasticity) as the indention force changes. Damping can reduce damage because the energy absorbed and converted to heat is not available for breaking bonds in the material.

We used an Atomic Force Microsocpe (AFM; NanoScope IIIa, Digital Instruments, Santa Barbara, CA) with an add-on force/displacement transducer (TriboScope, Hysitron Inc., Minneapolis, Minnesota). The Hysitron transducer held a polished diamond probe in place with capacitors that were used to sense the position of the probe and to impart vertical forces for indenting and imaging the specimen. Measurement regions were selected for minimal slope and surface topography as evidenced by the depth variation in AFM scans and the symmetry of the residual indents.

**Figure 2:**
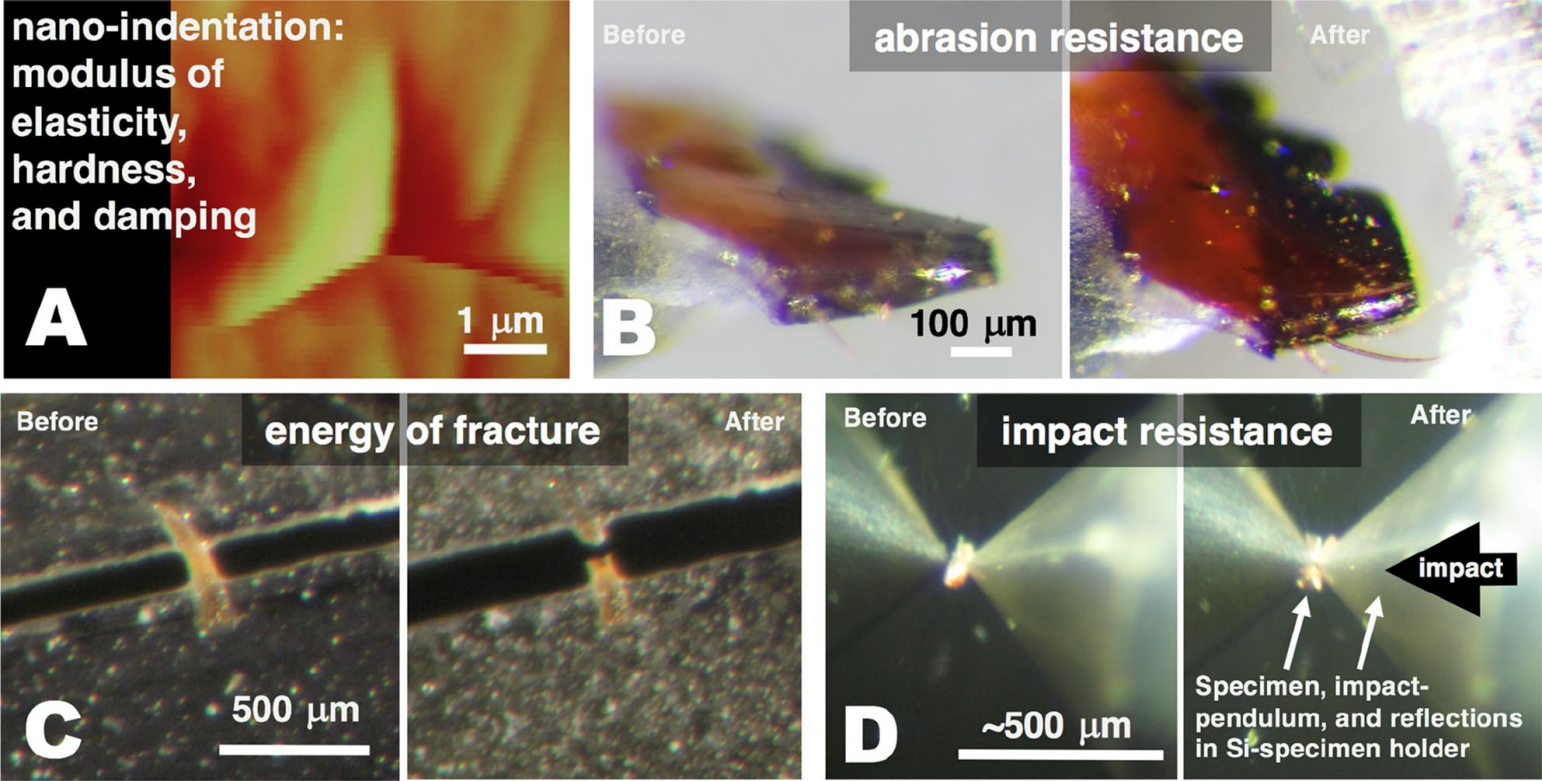
Images of testing samples for each of the measured properties. (**A**) AFM image of a residual indentation on the natural surface of the Mn-HEB region of the sting of a scorpion, *Hadrurus arizonensis*. Indentations were used in measuring hardness, modulus of elasticity and damping properties. Natural “scratches” are visible on the original surface around the triangular indentation made by the cube-cornered indenter. (**B**) Mandible of an ant, *Atta cephalotes*, before and after an abrasion testing session. The tip of the zinc-rich distal tooth in the “before” image has been flattened during a preliminary abrasion session. (**C**) Images from before and after energy of fracture testing of a 12 μm thick Zn-HEB test piece made from the nearly-flat side-surface of the fang of a spider, *Araneus diadematus.* The original fang shape is evident with the tip of the fang towards the top and the proximal side to the left. (**D**) Images from before and after impact resistance testing of a 12 μm thick Zn-HEB test piece made from the fang of a spider, *Araneus diadematus.* The test piece covers a 50 μm diameter backing-pit milled (using FIB-SEM) into a (reflective) silicon chip. The piece has been shattered from the impact in the image on the right.

In order to make the most biologically relevant measurements, indentations were made on regions of the external surface of structures that directly contact the environment. However, we also made measurements on cross-sections of the arthropod “tools” as part of preliminary SEM and indentation investigations to ensure that the thickness of epicuticle or other surface material would not distort the results for HEBs. Figure 2A shows an indentation on an un-polished surface—the original surface topography is visible as linear scratches that are small compared to the pyramidal indentation. When we could not avoid indentation-scale topography, we hand-polished the surface with 2000–12,000 grit sandpaper (Micro-mesh sheets, http://micro-surface.com), to smooth the surface on the scale of the test indents.

Specimens were mounted on atomic force microscopy (AFM) specimen disks (TedPella Inc., Redding, CA) in a mound of epoxy composite. The composite was prepared by mixing approximately 0.45 g of 400-grit aluminum oxide powder (Buehler, Evanston, Ill., Ted Pella Inc., Redding CA, or Kramer Industries, Piscataway, NJ—the later preferred because it was less reflective) with 0.075 mL each of resin and hardener (Quick Set Epoxy; Loctite, Rocky Hill, CT, and 5 m Quik-Cure Epoxy, Bob Smith Industries, Atascadero CA). The composite was stiff enough that the unpolished specimen had to be pressed in and could be oriented before curing so that the desired indentation region would retro-reflect a light beam sent through the eyepiece of a dissecting microscope back into the microscope, ensuring that the desired region would be flat for AFM scanning and indentation. The mounted specimens were placed in an oven at 39 °C for at least 1 h to cure the epoxy composite. Samples that were not tested immediately were kept on moist paper in a container in a refrigerator and were tested within 24 h to avoid dehydration and other changes. Additionally, the epoxy composite served as a barrier to reduce loss of water through the cut surfaces.

The epoxy composite mounting technique was tested to ensure that small (about the same size as the biological specimens) "floating" glass cover-slip pieces would yield the same hardness and modulus of elasticity values as large, flat-mounted cover-slip pieces, and re-checked when relevant products were changed.

In order to test whether there were any rapid changes in hardness or modulus of elasticity of HEBs, we tested a *H. arizonensis* sting, that was mounted for AFM measurements while still attached to an anaesthetized scorpion. We did not find a significant trend with time for 14 measurements made between 10 or 20 min (Zn-HEB and Mn-HEB respectively) after separating the live scorpion from the sting, and 5 h (largest R^2^: 0.006). These fast results were also comparable to results made using the standard technique, indicating that the technique described above was sufficient to prevent significant changes from dehydration. We also made preliminary measurements on scorpion joint cuticle, armour teeth, and other non-HEB regions of the cuticle, and found none that were harder than the region at the base of the sting, used here to represent non-HEB cuticle.

We used a pyramid-shaped diamond probe with cubic corner facets (90° between the three faces)^14,31^. The steeper angle of the cubic tip, relative to a more commonly used Berkovich tip, made it easier to avoid surface features such as hairs. The diamond probe was positioned on the specimen using a 30× extra short focus monocular (M1030, Specwell Corporation, Tokyo, Japan). The indentation sequence began with the force being ramped linearly from 0 to 2 milliNewtons (mN) in 0.1 s, maintained at 2 mN for 10 s. The force was then ramped down to 1.5 mN over 0.1 s and then the force was varied sinusoidally at 10 Hz (for 25 cycles) with a peak-to-peak amplitude of 1.0 mN, in order to measure dynamic properties. The force was then ramped to 0 in 0.1 s.

#### Probe-extension (Oliver–Pharr) and image-based measurements

Two methods of obtaining the modulus of elasticity and hardness were employed^14^. In the first method, values were obtained only from force–displacement curves using the Oliver–Pharr technique^52,53^. The modulus of elasticity was obtained from the slope of the force–displacement curve at the beginning of withdrawal of the indenter. Oliver–Pharr hardness values were calculated from the intercept of this sloped line with the line of zero force.

In addition to the hardness value obtained from the force–displacement curves, we also obtained a hardness value based on measurements of the size of the residual indentation. These image-based hardness values were calculated as H = F/A, where “F” is the maximum force applied to the probe, and “A” is the projected area of the residual indentation, obtained from the perimeter of the indentation measured on an AFM image (e.g. Fig. 2A) made by scanning the indenting probe itself minutes after indenting the specimen.

The Oliver–Pharr method is inaccurate if the indentation force causes the surface of the specimen to move, such as for improperly backed specimens, because it assumes probe extension is a measure of indentation depth. In contrast, the image-based method is nearly insensitive to global displacements of the specimen because it is based only on the applied force and measurements of the residual indentation. We found it useful to obtain both values to check each other: on several occasions differences between the two measured values indicated support problems. This is important for biological specimens with multiple layers, voids, etc. For example, if there is a lumen under the shaft but not the tip, the tip may appear harder because it displaces less. In addition, calibration problems, such as from fractured silica, were quickly identified by differences in the image and Oliver–Pharr values. Finally, the image method is not sensitive to other artifacts, such as “pile-up”, that are associated with estimating contact region from probe extension^53–56^.

There is a potential difference between the Oliver–Pharr and image hardness values associated with the different time scales. The Oliver–Pharr hardness is measured during the probe withdrawal while the image of the residual indent is obtained a couple of minutes after indentation. If the indent partially recovers in the interim, the image technique would be based on a smaller residual indentation resulting in a higher hardness value. We prefer the image method not only because it is robust to imperfectly supported specimens, but also because we would like our hardness measurement to reflect the long-term indentation damage done to the tips and blades. To test that the indent size had stabilized by the time we measured it, we re-measured an indentation in the zinc-region of a scorpion sting after more than 6 months and found that the indentation diameter had decreased little, by about 15%.

We also calculated an image-based modulus of elasticity value that has been suggested for materials that produce “pile-up” artifacts^53^. The area measured from the image of the residual indentation (used for the image-based hardness), was substituted for the contact area calculated from probe extension in Eq. (6) of Oliver & Farr, 2004 ^53^.

**Figure 3:**
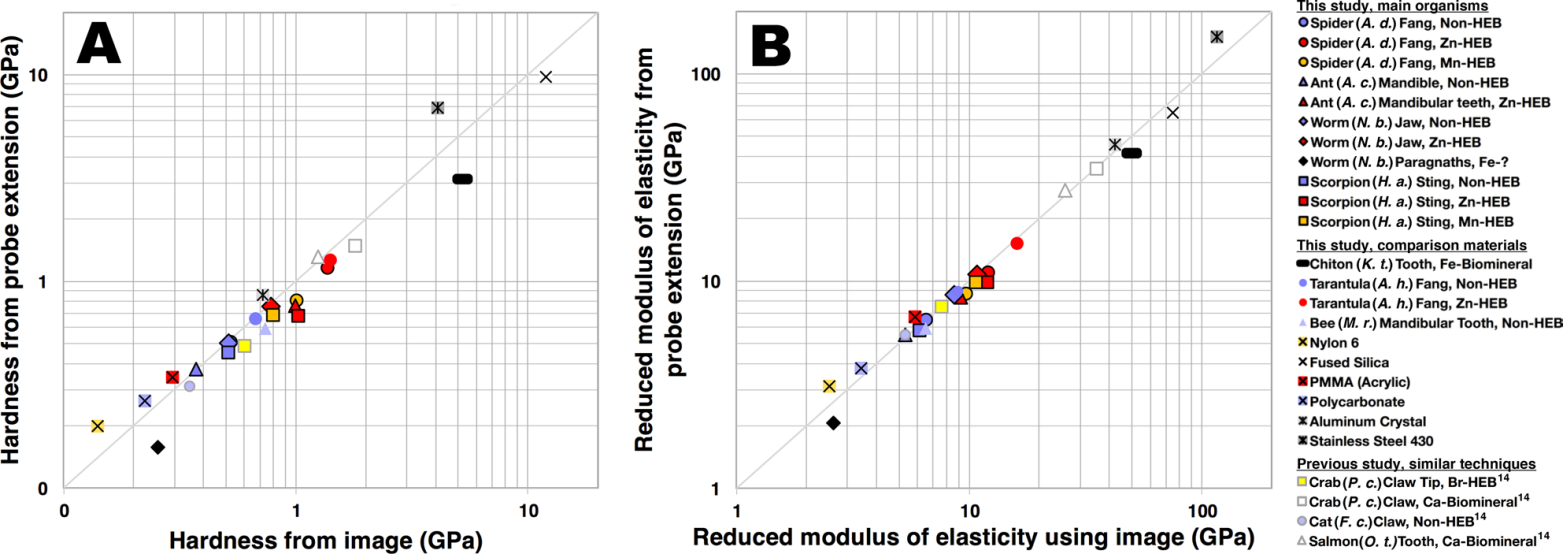
Comparison of probe-extension (Oliver–Pharr) and image-based techniques for hardness (**A**) and reduced modulus of elasticity (**B**) for our indentation data. Pile-up may account for higher (above the line) Oliver–Pharr values of some of the metals and plastics.

Notwithstanding the differences in image-based and probe-extension based (Oliver–Pharr) measurements, there was little practical difference in results, as shown in Fig. 3A,B. The metals and plastics that we measured for Fig. 3 tended to have slightly higher Oliver–Pharr hardness values than image-based values, possibly because of “pile-up” artifacts.

In the “Results” section, we plot the values obtained using the images, but the Oliver–Pharr values are included along with image-based values in the results table, Table 1.

#### Loss tangent

Dynamic mechanical properties were measured from the sinusoidal segments of the indentation sequence by comparing the amplitude and phase of the displacement to the applied sinusoidal force^57^. Phase lags associated with the transducer and electronics were determined assuming zero true-lag from a fused silica standard obtained using the same indentation sequence and force. The loss tangents obtained in this way were for the high-stress regimes associated with indentation, as compared to low-stress tests involving bending without plastic deformation.

#### Calibration for Oliver–Pharr measurements

In order to obtain the contact area from the indent depth, the shape of the indenting tip must be known. We characterized the indenting tip shape directly using scanning electron microscope images, so that we could make deep, micron-scale indents that would be evident on un-polished biological surfaces. We could not make indents in silica as deep as the indents desired for our biological specimens without fracturing the silica (fracture for the cubic cornered tip began at about 3 mN) so we could not use the usual technique of estimating the tip shape at depth by calibrating with fused silica. The tip shape was characterized by three measurements: first, the angle of the three-sided pyramidal tip (*α*), second, a measure of the bluntness of the tip (*B*), the distance between the apex of the tip if it were an ideal pyramid and the actual blunt tip, and third, a measure of the distance from the blunt tip beyond which the shape of the tip was not distinguishable from an ideal pyramid (*I*). The value “*I*” was used as a limit: only indents with greater depth than “*I*” were used to calculate mechanical properties. For these deeper indents, the following description of the projected area (*A*) of the contact region between the tip and the specimen was used:

$$A= \frac{(0.433)(4){(D+B)}^{2}}{\frac{1}{{\mathrm{tan}}^{2}\left(\frac{\alpha }{2}\right)}-0.3333},$$

where *D* is the depth of the indent, determined by the extension of the indenting probe, 0.433 is the ratio of the area of an equilateral triangle to the square of the length of a side, and 0.3333 is tan^2^ (30°). As an example, the tip used for the majority of measurements was characterized by *α* = 89.9°, *B* = 115 nm, *I* = 100 nm. Thus, for indents with a depth greater than 100 nm, for our tip, *A* = 2.58 (*D* + 115 nm)^2^.

#### Measurement of residual indentation area

The area of the residual indent was measured using an AFM image, obtained minutes after the indentation, using the indenting tip as the imaging tip. To minimize inaccuracies in indent perimeter determination, caused by finite size of the imaging probe or other systematic errors, we calibrated our area measurements so that we obtained a median value of 70 GPa for measurements of the modulus of elasticity on fused silica. Because there is some subjectivity in measuring the size of the indentation, operator-specific calibrations, based on each operator’s measurements of fused silica, were used for most of the measurements.

### Test piece preparation for impact resistance and energy of fracture measurements

We measured resistance to impact and fracture using custom miniature versions of testing devices that fracture or damage standardized “test pieces” of materials. We prepared test pieces as follows: the fresh (usually immediately after removal from the organism) specimens were adhered to one end of a glass slide using a marine epoxy (Loctite, Rocky Hill, CT) which required a curing time of 2 h at 39 °C, or with cyanoacrylate adhesive (Krazy Glue, all purpose, Elmer's Products)^14^, which required no extra curing time. A flat region (> 100 μm diameter, but not wide enough to reduce the thickness of the HEB region in the center to less than 12 μm) was polished on a specimen by grinding the slide with the specimen against a sequence of flat 2000, 6000 and 12,000 grit sandpaper (Micro-mesh sheets, http://micro-surface.com). The specimen was removed from the adhesive using a scalpel, inverted, and the polished-flat region was adhered to the glass with a thin film of water and surrounded by a small bead of marine epoxy. The water film kept the epoxy from getting pulled under the specimen by capillary action. The epoxy was cured and the specimen polished to a thickness of 12 ± 2 μm as determined with a digital micrometer, using the same sandpaper sequence. The resulting test piece was then freed by scraping the epoxy from around the edges using a scalpel blade. The area of the pieces varied according to the size of flat regions and was, typically, hundreds of microns on a side.

Maintaining hydration was especially important for these test pieces because they were only 12 μm thick and so they could dry quickly. To reduce artifacts from drying or other changes in the tested material, all preparation and testing took place in a ~ 15 m^3^ enclosure maintained at greater than 90% relative humidity.

Although we used FIB-SEM (Focused Ion Beam-Scanning Electron Microscope) to shape specimens of the materials for molecular fragment analysis, we did not use this technique for preparing our micron-scale test pieces for several reasons: potential material property changes caused by beam damage, subjection to vacuum, and because some of the test pieces needed to be large enough that they would be difficult to make with FIB-SEM.

### Impact resistance measurements

A custom testing device was built to compare the energy required for a swinging pendulum to shatter test pieces of the different materials (Fig. 2D). A 12 ± 2 μm thick test piece was adhered by the moisture in the high-humidity enclosure and held in place with an adhesive (spots of cyanoacrylate, Krazy Glue, all purpose, Elmer's Products, www.elmers.com, or 5-min epoxy gel) over a 50 μm-diameter circular pit milled in a silicon wafer using a FIB-SEM apparatus. The pendulum, made of carbon fiber and aluminum (length: 0.2 m, moment of inertia: 4.25 x 10^−6^ kg m^2^) with a diamond impactor tip polished to a diameter of 20 μm, was held by miniature bearings and electronically released from increasing heights until the test piece fractured. The energy required to fracture the specimen was calculated from the release height from which the pendulum fractured the test piece. This energy was normalized by the measured thickness of the specimens to give joules required per meter of thickness. Nevertheless, we consider this test to be a relative test that is not expected to be generalizable to all impacts, as the energy to fracture is likely to depend not only on thickness but also on variables such as the diameter of the impactor tip and the diameter of the backing hole.

This impact test differs from Charpy and Izod tests in that the energy required to fracture the specimen was measured by releasing the pendulum from increasing heights until the specimen fractured, rather than by releasing it from a height sufficient to fracture all specimens, and measuring the residual energy of the pendulum after impact. The advantage of our threshold technique is that the threshold of fracture is likely the biologically important quantity, and direct determination of the fracture threshold avoids the possibility that the energy deposited in a single highly-energetic impact might be partially expended in plastic deformation, leading to an overestimate of the threshold energy. A drawback of our technique is that impacts that do not break the specimen may produce damage that weakens the specimen for subsequent impacts. Nevertheless, all specimens were subjected to the same series of increasingly energetic impacts, until fracture, and were thus comparable.

### Energy of fracture measurements

We measured the energy or work required to slowly break a test piece in two (Fig. 2c) using a custom fracture toughness measuring device^14^. The device drove apart two microscope cover slips bridged by the test piece until it split in two, while recording the required force and the displacement (work is the product of force and incremental distance). This work, divided by the area of the new post-fracture surfaces, is the energy of fracture, reported in Joules per meter squared. It is a measure of the energy required, per unit area, to break the bonds that originally held the two pieces together (as long as the kinetic energy is relatively small—the pieces do not fly away), and is one indication of the resistance of a material to fracture.

The length of the fracture was measured using a microscope and multiplied by the thickness of the test piece to obtain the fracture area. This area was used to normalize force–displacement curves. The work of fracture per unit area of the fracture was obtained by numerically integrating these normalized force–displacement curves. The load cell was designed to be stiff in order to minimize storage of energy within the apparatus as the specimen underwent tension^58^. Fracture planes were perpendicular to the original surface and approximately perpendicular to the long axis of the “tool” (Fig. 1C).

The test protocol was altered from that used previously^14^ because the test pieces used here were smaller. Test pieces were not notched in order to avoid fractures from the notching process, and the specimens were adhered to the test apparatus in place (Krazy Glue, all purpose, Elmer's Products, www.elmers.com) to avoid premature fracture. To improve the bonding of the cyanoacrylate adhesive to the glass cover slips in the high humidity atmosphere, we treating the cover slips with a 10-s dip in a 2% (by volume) 3-aminopropyltriethoxysilane (Sigma Chemical Co., www.sigmaaldrich.com), 98% acetone solution, followed by rinses in deionized water and air drying. The test protocol for the ant mandibular teeth varied from the others in that whole teeth were fractured instead of 12μ polished test pieces.

A consistency test with AFM data was developed to identify cases of imperfect bonding to the cover slips, when part of the measured energy was expended in partially pulling the test piece out of the cyanoacrylate adhesive. For samples subject to this problem (usually specimens with small adhesive contact areas, such as the fang specimen in Fig. 1C), we required that the force–displacement curve be consistent with the stiffness of the test piece, expected from a model based on the shape of the individual test piece and the slope of the force–displacement curve for the insertion portion of the nano-indentation sequence for that material. When a piece partially pulled out and failed this test, the apparent stiffness was much lower than the expected stiffness (from nano-indentation) and slight stretch marks were often visible in the adhesive on close inspection.

This stiffness consistency test was also found to be useful in identifying cases where part of the fracture was pre-existing but had not been visible in the test piece. The pre-existing fracture would tend to reduce the effective width of the specimen and thus could be identified by a lower than expected stiffness under tension.

### Abrasion resistance measurements

We measured the energy required to abrade away a volume of material from our specimens by holding them against a rotating abrasive disk. The energy used in eroding the material is given by the force of friction multiplied by the distance traveled over the abrasive paper (work is the product of the force and incremental displacement), with units of Joules per meter cubed of volume worn away.

The "pin on disk" type testing device, developed for testing pieces of crab cuticle^14^, was used with modified procedures for the smaller specimens here. Instead of cylindrical core samples, whole, approximately conical tips of teeth, fangs or stings were used (Fig. 2B). The samples were affixed with cyanoacrylate gel adhesive (Maxi-Cure, Bob Smith Industries, Atascadero CA) to a steel pin held in the head of the wear tester. This head was mounted on a custom-made load cell that measured the horizontal force produced by friction between the specimen pin and the abrasive turntable. During the wear test, the specimen pin was held against the turntable with adjustable weights that, for the standard test, produced a downward force of 0.019 Newtons. The surface of the turntable was covered with 600 grit abrasive paper (#413Q, 3 M Corporation, www.3M.com). The turntable rotation period was usually set to about 4 s, resulting in an interaction velocity of 0.027 m/s.

The volume worn away was calculated from “before” and “after” measurements of microscope images (image J software) taken from the side (e.g. Fig. 2B) to measure the height of the approximate cone of worn material, and from face-on in order to measure the area of the base and top of the frustum of worn-away material. The horizontal force was recorded continuously during the wear period. The wear rate (*w*), defined as the volume worn away per unit energy expended, was approximated as follows:

$$w= \frac{V}{Fd} ,$$

where *F* is the average force of friction measured during the wear period by the load cell, *d* is the distance traveled by the pin over the abrasive paper and “*V*” is the worn volume, approximated as a frustum:

$$V = 1{/}3\,\Delta L \, (A1 + \left( {A1*A2} \right)^{1/2} + A2)$$

where “*A*1” and “*A*2” are the areas of the worn surface before and after the wear sessions (the area of any voids or internal lumens was subtracted from the area of the cross sections) and Δ*L*, the change in the length of the specimen due to wear. We defined wear resistance as the inverse of the wear rate, *1/w*. While we expect this test to be most useful for relative comparisons, and the value is expected to vary somewhat with abrasive properties and normal forces, we found no statistically distinguishable difference in values from 4 samples that were re-run using a ten-times greater force to press them against the abrasive paper^14^.

### Molecular composition and nanometer-scale structure

In order to better understand the composition and structure of the HEBs—down to an atomic scale—we examined a representative HEB using Atom Probe Tomography (APT). We checked the APT results and studied their generality using Time-of-Flight–Secondary Ion Mass Spectrometry (ToF–SIMS). Both of these techniques use a pulsed beam (laser and ion respectively) to break the specimen into molecular fragments that are accelerated to a detector; for a particular charge, heavier fragments travel more slowly and arrive later at the detector. The arrival time differences are used to identify the fragments by their mass, giving information about, for example, the atoms attached to zinc atoms in the specimen and, from APT, the spatial distribution of zinc atoms on a nanometer scale.

### Atom probe tomography (APT)

APT is a 3D nanoscale characterization method in which field evaporated ions from a sharpened needle specimen are analyzed by a position-sensitive single-particle detector, in order to provide an isotopically resolved three-dimensional representation of the real-space specimen elemental distribution^59^. The field evaporation of non-conductive samples is achieved using a pulsed laser focused on the needle specimen apex.

A FIB-SEM based lift-out procedure was used to prepare needle-shaped APT specimens using FEI Helios 600i at the University of Oregon CAMCOR facility, and a Helios Dual Beam Nanolab 600 FIB-SEM housed at Environmental Molecular Sciences Laboratory, PNNL.

The APT analysis was carried out using a CAMECA LEAP (local electrode atom probe) 4000X HR system equipped with a 355 nm wavelength picosecond pulsed UV laser. A 30 K sample base temperature and a 100 or 200 kHz laser pulse repetition rate was used. Atom probe data reconstruction and analysis was performed using Cameca IVAS software.

#### *Development of APT techniques for these organic materials*.

APT has not typically been used to examine organic materials, so we began by examining standards (such as zinc picolinate) and adjusting beam current densities and other parameters in both the FIB-SEM preparation of APT samples and in APT itself in order to minimize physical damage detected with SEM and to minimize differences between the chemical formulae and APT results for standards^60^. We found that current densities often employed in FIB-SEM milling were much too high for our organic specimens, resulting in beam damage visible in SEM.

Based on the standards and SEM evidence of damage, we used FIB-SEM currents for producing the sample needles, and APT laser pulses for promoting evaporation, that were similar to or smaller than those used in other investigations of organic materials^61–68^. We used ≤ 21 pA for the electron beam, ≤ 80 pA for ion beam “trenching”, 7.7 pA for ion beam imaging and for cutting the cantilever “liftout” piece, and ≤ 24 pA for sharpening needles. The results reported here are based on samples analyzed using 10, 20 or 100 pJ laser pulses.

Identification of molecular fragments from mass-to-charge ratios is particularly difficult for organic materials because of the many possible fragments of similar mass, and several techniques have been developed to aid in this analysis^61–64,68–73^.

Our identification of zinc-containing fragments was simplified by the pattern of the 3 or 4 main zinc isotopes (Fig. 6A). In addition, we used resources with lists of fragments as a function of mass (e.g. https://webbook.nist.gov/chemistry/mw-ser/). We also cross-checked fragment identification using a Time-of-Flight–Secondary Ion Mass Spectrometry (ToF–SIMS) system.

### Time of flight–secondary ion mass spectrometry (ToF–SIMS)

We used a ToF–SIMS system that had a higher mass-to-charge resolution than the APT system (although it had micron- instead of nanometer-scale spatial resolution) in order to check APT fragment identification. The higher mass sensitivity of the ToF–SIMS system provided additional evidence that, for example, the fragments identified as ZnCN were not actually ZnC_2_H_2_, which is only about 0.02% lighter. We also used ToF–SIMS to study the larger-scale spatial distribution of fragments, and similarities with HEBs from other species.

We used an ION-TOF ToF–SIMS IV, manufactured by ION-TOF GmbH, Muenster, Germany. The primary ion beam was Bi^3+^ (25 kV, 10 kHz, 0.4 pA); the static limit (2 × 10^12^ ions/cm^2^) was not exceeded. The dimensions of the analysis area varied, but were between 100 × 100 μm and 300 × 300 μm. A low energy electron beam was used for charge neutralization. The spectra were analyzed using the vendor’s software. Chemical maps of peaks of interest were created from the total spectra and used as a basis for retrospective analysis—i.e., pixel-specific extraction of spectra in order to determine the chemical makeup of features of interest.

## Results

### Mechanical properties

Table 1 contains the results for Zn- and Mn-HEBs, non-HEB material, engineering materials, biomineralized materials, and bromine-HEBs, all measured using similar techniques.

**Table 1:** Results from mechanical property measurements of animal and engineering materials.

Material	Hardness (Gpa)					Modulus of elasticity (GPa)					Loss tangent, 10 Hz			Energy of fracture (J/m^2^)			Impact resistance (J/m)			Abrasion resistance (J/m^3^)		
	Mean, image method	s.d.	Mean, O–P method	s.d.	n	Mean, image method	s.d.	Mean, O–P method	s.d.	n	Mean	s.d.	n	Mean	s.d.	n	Mean	s.d.	n	Mean	s.d.	n
**Leafcutter ant (*Atta cephalotes*)**																						
Mandible, HEB-free region	0.37	0.05	0.38	0.05	99	5.3	1.1	5.50	1.10	99	0.041	0.016	24	***3885***	***2514***	***8***				4.3E + 09	3.5E + 09	27
Manibular teeth, Zn region	0.99	0.43	0.77	0.14	82	9.2	2.2	8.36	2.01	82	0.069	0.031	26	546	151	4				5.0E + 09	2.9E + 09	13
**Spider (*Araneus diadematus*)**																						
Fang, HEB-free region	0.52	0.14	0.51	0.07	55	6.5	0.9	6.52	0.81	55	0.035	0.023	26	697	700	8	3.7	1.7	10	2.4E + 09	1.4E + 09	17
Fang, Zn region	1.37	0.38	1.17	0.30	56	12.0	2.4	11.10	1.91	56	0.036	0.032	28	903	690	6	4.8	1.5	7	2.4E + 09	1.2E + 09	16
Marginal teeth, Mn region	1.01	0.28	0.81	0.09	45	9.6	1.5	8.74	0.92	45	0.029	0.009	21							4.0E + 09	8.8E + 08	7
**Nereid worm (*Neanthes brandti*)**																						
Jaw, HEB-free region	0.51	0.12	0.50	0.08	48	8.6	1.3	8.62	1.17	48	0.025	0.015	22	907	697	5	20.9	5.6	9	2.4E + 09	1.3E + 09	15
Jaw, Zn region	0.78	0.21	0.76	0.07	50	10.8	1.6	10.76	1.55	50	0.021	0.012	22	945	747	5	18.9	4.9	12	5.8E + 09	3.1E + 09	13
Paragnaths, Fe region	0.26	0.07	0.16	0.06	3	2.6	0.8	2.07	0.84	3	0.148	0.009	3									
**Scorpion (*Hadrurus arizonensis*)**																						
Sting, HEB-free reg	0.51	0.13	0.45	0.06	21	6.1	2.2	5.79	1.94	21	0.002	0.006	21	2981	1641	6	13.4	3.3	5	2.7E + 09	7.7E + 08	4
Sting, Zn region	1.02	0.35	0.68	0.15	27	11.9	2.3	9.94	1.71	27	0.054	0.029	24	548	661	2	11.4	4.9	6	5.7E + 09	2.0E + 09	5
Sting, Mn region	0.80	0.22	0.69	0.10	38	10.6	1.7	9.93	1.00	38	0.021	0.013	32	180	298	5	9.1	5.6	12	5.2E + 09	3.2E + 09	15
**Salmon (*Oncorhynchus* sp.)**																						
Teeth, Ca biomineral region	1.24	0.12	1.31	0.12	6	25.8	2.5	27.46	1.70	6	0.072	0.006	3	1706	751	9				2.9E + 09	2.5E + 09	9
**Chiton (*Katharina tunicata*)**																						
Radula, Fe biomineral region	5.21	4.02	3.14	1.28	29	49.5	23.2	41.48	15.87	29	0.198	0.081	20	20	22	4				2.9E + 10	1.3E + 10	9
**Chiton (*Cryptochiton stelleri*)**																						
Radula, Fe biomineral region	6.27	1.07	2.48	0.11	3	71.1	15.7	45.40	12.83	3	0.231	0.010	3	6	3	3						
**Crab (P*achygrapsus crassipes*)**																						
Claw, Ca biomineral region	***1.79***	***0.35***	***1.49***	***0.40***	***38***	***35***	***11***	***35***	***12***	***38***	***0.020***	***0.020***	***20***	***348***	***138***	***6***				***1.3E* + *09***	***3.1E* + *08***	***7***
Claw, Br HEB region	***0.60***	***0.08***	***0.49***	***0.12***	***45***	***7.6***	***2.2***	***7.6***	***2.7***	***45***	***0.130***	***0.070***	***19***	***2931***	***574***	***8***				***1.0E* + *09***	***2.0E* + *07***	***7***
**Cat (*Felis cattus*)**																						
Claw, HEB- and biomineral-free	***0.35***	***0.02***	***0.31***	***0.03***	***6***	***5.3***	***0.3***	***5.5***	***0.5***	***6***				***13,321***	***8810***	***9***						
**Tarantula (*Aphonopelma hentzi*)**																						
Fang, HEB-free region	0.67	0.11	0.66	0.03	6	9.0	1.3	8.91	0.57	6												
Fang, Zn region	1.41	0.28	1.27	0.14	4	16.0	2.9	15.25	2.36	4												
**Leafcutter bee (*Megachile rotundata*)**																						
Mand. teeth, HEB- and biomineral-free	0.74	0.26	0.59	0.16	8	6.5	2.5	5.95	2.38	8	0.110	0.006	5									
**Engineering materials**																						
Nylon 6 (not dried)	0.14	0.01	0.20	0.01	3	2.5	0.2	3.10	0.14	3	0.100	0.010	3							3.2E + 09	5.1E + 08	37
PMMA (acrylic)	0.29	0.06	0.34	0.04	17	5.9	0.7	6.74	0.83	17	0.071	0.007	6	188	152	4						
Polycarbonate	0.22	0.07	0.26	0.01	19	3.4	0.9	3.80	0.49	19	0.016	0.003	10				2.7	0.8	5			
Fused silica	11.99	4.78	9.80	0.93	73	75.1	16.0	64.68	12.90	73	- 0.001	0.025	73	3.1	3.5	5	2.4	1.1	11	1.2E + 10	5.9E + 09	9
Aluminum, single crystal	0.72	0.17	0.86	0.04	7	42.3	16.0	45.38	16.04	7	0.208	0.014	7									
Stainless steel, 430	4.09	0.56	6.90	0.32	7	116.4	12.0	151.43	12.30	7	0.224	0.013	7									

"O-P method" refers to the Oliver-Pharr (probe extension) method. Bold italic type indicates values from Schofield et al.^14^. The number of measurements is indicated by "n". A maximum of 3 measurements were made per specimen, so the number of individual "tools" sampled for a property was equal to or greater than n/3.

**Figure 4:**
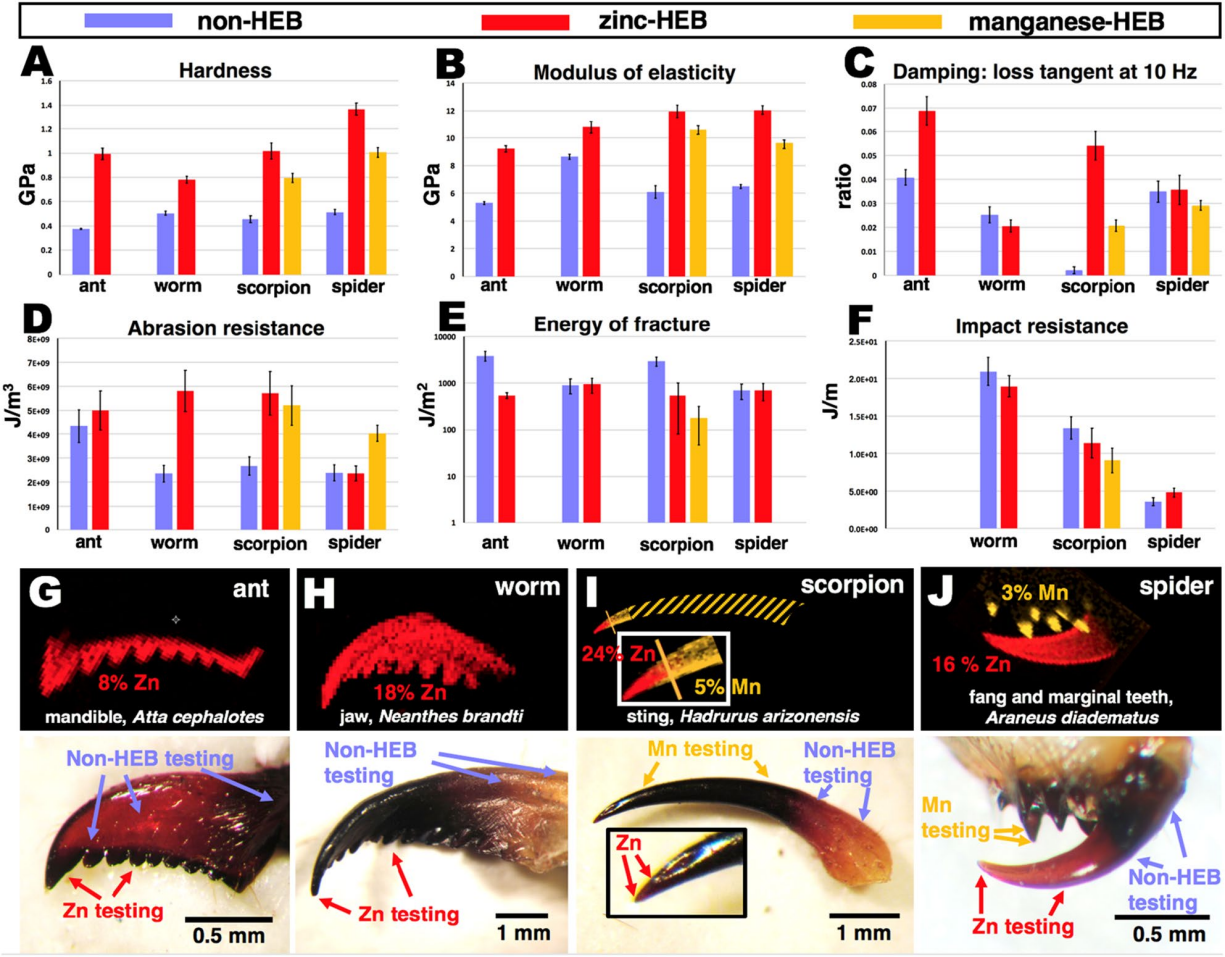
(**A**-**F**) Results from mechanical testing. Zn-, Mn- and non-HEBs, are indicated by red, orange and blue coloring, respectively. Error bars show the standard deviation of the mean of measured values, standard deviations are given in Table 1. (**G**–**J**) Images of the tested structures; top row, high-energy ion microprobe^12^ elemental maps of zinc (red) and, superimposed, manganese (orange). Bottom row, photographs of specimens of the indicated species. The element concentration values (percent of dry mass) are for the most enriched regions ^7,12,31,74^. The orange hash-marked region of the scorpion sting in “I” indicates the extent of the Mn-HEB region in the photograph below it, but is not part of the microprobe image, which is shown enlarged in the boxed inset. Regions used in mechanical testing are indicated with arrows in the photographs, except that the joint of the ant mandible (just outside the right-hand side of the photograph) was used for non-HEB abrasion testing. Because of the different scales of the tests, there may be differences in the material sampled: micron scale indents were used for modulus of elasticity, hardness and damping measurements; 12 μm thick surface layer pieces were used in energy of fracture and impact testing, and even larger regions were used in wear testing (see Fig. 2). Links to videos showing these “tools” in action: (1) Ant, *Atta cephalotes*, https://vimeo.com/453712530/71d8ec61c7. (2) Nereid worm, *Neanthes brandti*, https://vimeo.com/453715976/818fe6f0be. (3) Scorpion, *Hadrurus arizonensis*, https://vimeo.com/453714558/1831b89eac. (4) Spider, *Araneus diadematus*, https://vimeo.com/453717522/30016da919.

**Figure 5:**
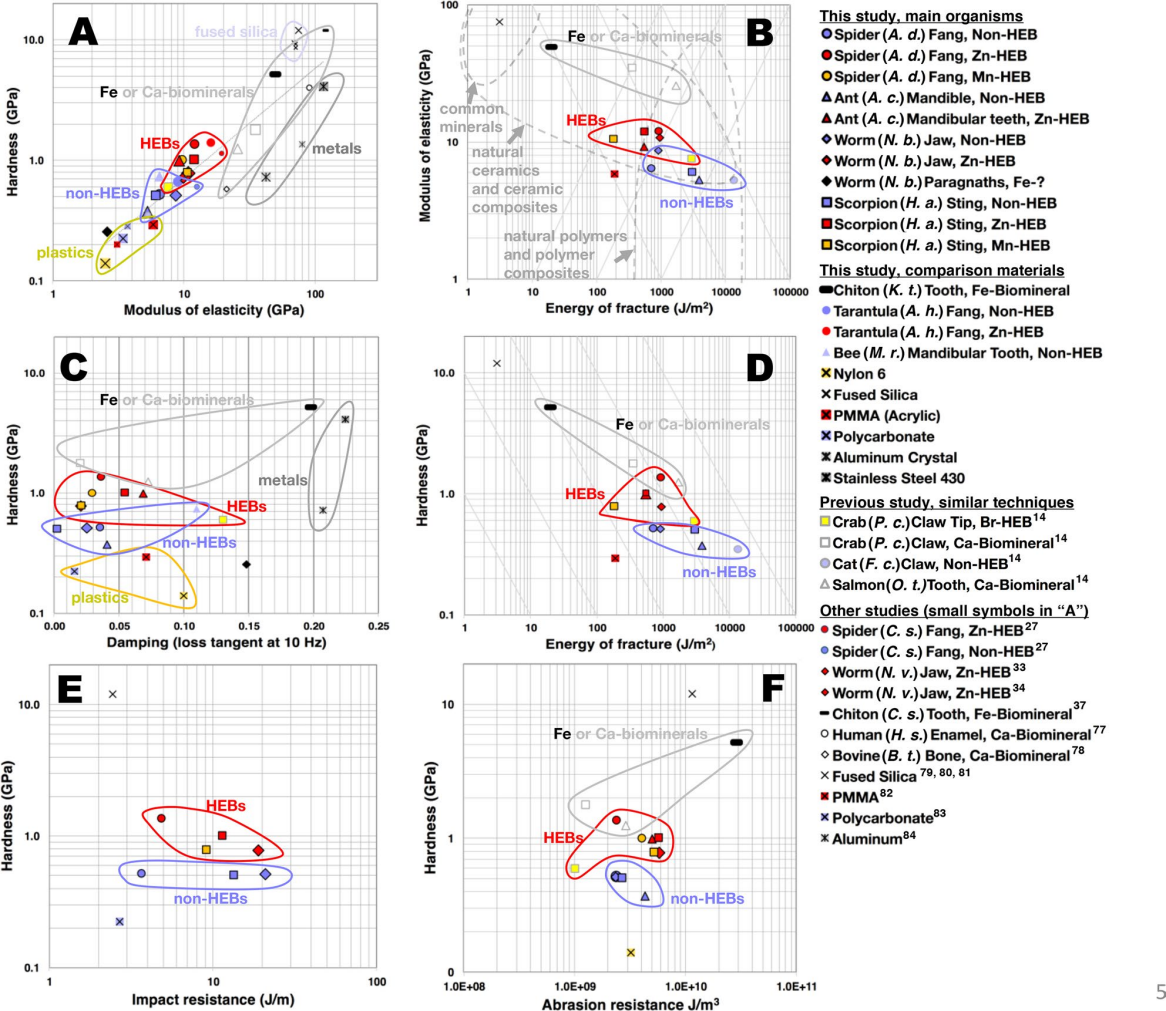
(**A**–**F**) Graphs of pairs of material properties, showing the means from Table 1 for HEBs, non-HEBs, biomineralized tissues, and engineering materials tested using the protocols here (large symbols, black outline or crosses) and in Schofield et al. ^14^ (large symbols, grey outline). The hardness and the reduced modulus of elasticity values were obtained using the image-based techniques described in the experimental section, except for the values from other groups, shown with smaller symbols and only in (**A**), which were made using the probe-extension (Oliver–Pharr) technique and made on dried or re-wetted specimens. Our image- and probe-extension based techniques yielded similar results (see Fig. 3), validating the comparison. Ashby’s^91^ material classes are indicated with dashed grey lines in (**B**). The susceptibility to fracture from a force challenge is approximately equal along the downward sloping lines in (**B**) (product of modulus and energy of fracture are equal) and to a displacement challenge along the upward sloping lines (the ratio of elastic modulus and energy of fracture are equal)^92^. The spider and worm Zn-HEBs (red circle and diamond respectively) have a slightly higher energy of fracture than their blue non-HEB counterparts, and the ant and scorpion Zn-HEBs (red triangle and square) and non-HEBs are along a line of equal susceptibility to a force challenge. In addition, the Zn-HEBs had similar or greater damping in (**C**) and abrasion resistance in (**F**) as compared to their non-HEB counterparts. The Fe-biomineral in the chiton tooth was harder and more resistant to wear, but took less energy to fracture. The Ca-biominerals had a higher modulus of elasticity, required slightly more energy to fracture, and were slightly less resistant to abrasion.

Figure 4 shows micrographs of the examined “tools” of the main ant, nereid worm, scorpion and spider species. Figure 4 also indicates the HEB regions, metal concentrations, and measurement locations, and the mean and standard error of the mean (SEM) measurement values for each property. Because of difficulties associated mainly with small size, not all properties were measured for all materials. All hardness and modulus of elasticity values in Figs. 4 and 5 are for image-based measurements, but the values were similar to the Oliver–Pharr values (see Fig. 3), and would not have substantially altered the conclusions below. The Oliver–Pharr values are included with image-based values in Table 1.

#### Hardness and modulus of elasticity in compression

Figure 4A (hardness) and Fig. 4B (modulus of elasticity in compression) show that, in all cases, the Mn- and Zn-HEBs were harder (smaller permanent indentations) and had a higher modulus of elasticity (stiffer structures) than the non-HEB materials in the indicated adjacent regions. The lowest hardness and modulus of elasticity values for both the Zn- and Mn-HEBs were significantly higher than the highest value of the tested non-HEB material in any of the organisms, even across phyla (hardness: p < 2 x 10^-10^; modulus of elasticity: p < 0.004).

#### *Damping (loss tangent or tan (*δ*)) at 10 Hz*

The loss tangent (tan(δ)), is a measure of damping or conversion of vibration energy to heat, which makes the energy unavailable for breaking bonds and increasing fracture area. The energy lost as heat during a sinusoidal indentation is proportional to the loss modulus while the energy stored is proportional to the storage modulus, and the ratio of the two is the loss tangent. Greater values of loss tangent indicate greater viscoelasticity, damping of vibrations and greater attenuation of propagating waves^57,85,86^.

Figure 4C shows that for both the ant teeth and scorpion sting tip, the indentation-based loss tangents were significantly higher for the tested HEBs than for the non-HEB cuticle (p < 0.00023). For the spider and worm species, the loss tangents were not significantly different for the HEBs and non-HEBs. However, as discussed below, all HEBs performed well when the “trade-off” between hardness and loss tangent^57^ is considered.

The loss tangent of the base of the scorpion sting was notably lower than for other materials, indicating that little of the stored energy was lost. This may be advantageous if the stored energy can be released to help puncture a target. The low damping in the thicker material at the base of the sting suggests the possibility that the scorpion sting stores energy in its base as it bends when it is pressed against the target, energy that is released to expand the developing fracture at the initiation of puncture. The higher damping and modulus of elasticity along the shaft of the sting, the Mn-HEB region, may help to reduce energy storage in a region of the sting that is more susceptible to fracture because its diameter is smaller than that of the base.

#### Abrasion resistance

Four of the six HEBs were significantly more abrasion resistant in our test (up to a factor of 3-times more) than the comparison regions (Fig. 4D); the ant mandible and spider fang did not differ significantly from comparison regions. The average abrasion resistance of the six HEB regions was significantly greater (p = 0.02) than the average abrasion resistance of the comparison regions.

#### Energy of fracture

Figure 4E shows that the energy required to fracture the HEBs was either about the same or significantly lower than for the tested non-HEB region of the same “tool”. A lower energy of fracture (greater susceptibility to fracture) might be expected a-priori, since the HEBs have a higher modulus of elasticity and there tends to be an anti-correlation, a trade-off, between modulus of elasticity and energy of fracture, especially in biological materials^85^. In the section below on correlated properties, we show the performance of the HEBs in this trade-off.

#### Impact resistance

Figure 4F shows that the impact resistance tended to vary more between species than between HEBs and plain material. The differences between HEBs and non-HEBs within an organism were also not consistent, suggesting that other differences in the materials had a greater effect on impact resistance.

### Structural and chemical properties: the homogeneity scale and the Zn binding site

The scale of the constituent “building blocks” may be a biologically relevant material property if the material is a composite and the grain size is large enough that it limits the sharpness of tips and blades and the precision of mated tool surfaces. The binding chemistry of the heavy elements is important in understanding the source of mechanical property differences.

**Figure 6:**
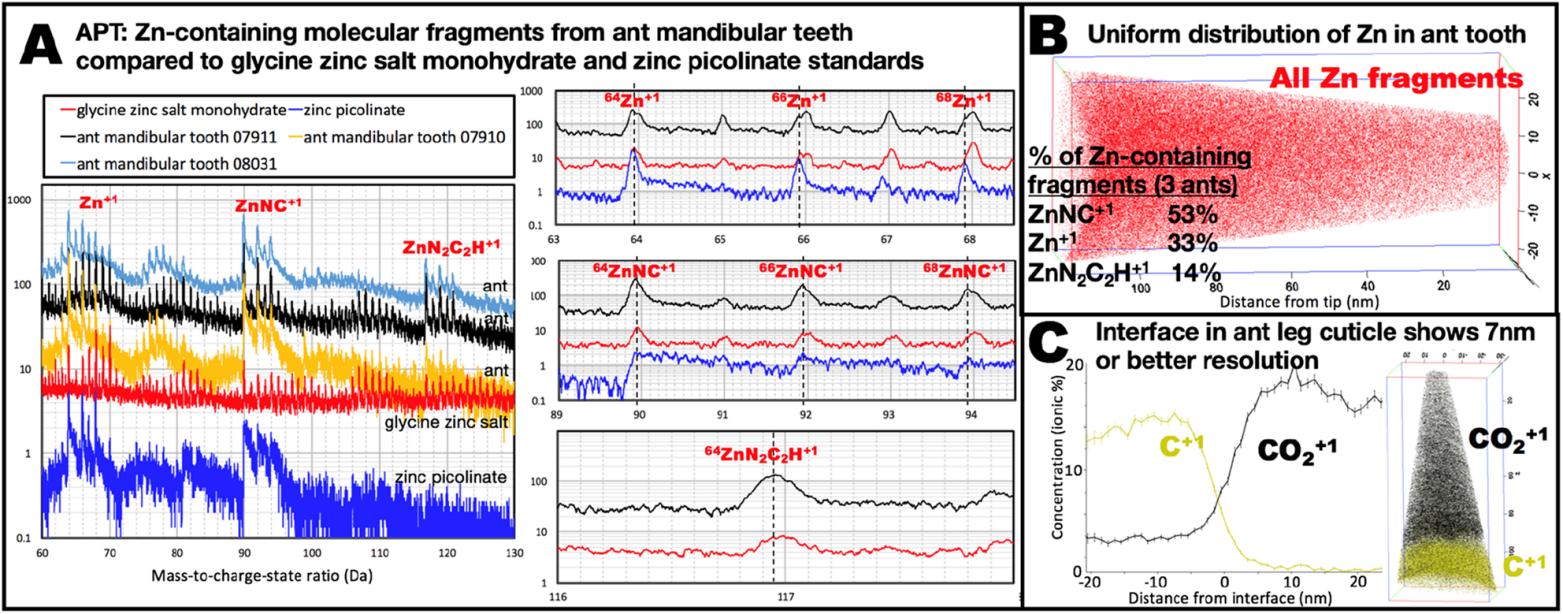
Atom Probe Tomography data for the Zn-HEB in the mandibular teeth of the ant *Atta cephalotes,* and standards. (**A**) Mass-to-charge-state ratio for molecular fragments of ant mandibular teeth and for standards that may have similar zinc binding. For the same charge, heavier fragments travel more slowly and appear further to the right in these time-of-flight based spectra. The plot at the left shows an overview of the spectral region with all of the identified molecular fragments containing zinc (Zn^+1^, ZnNC^+1^, ZnN_2_C_2_H^+1^). It shows the spectral similarity of different regions of an ant tooth (specimen 07910 and 07911) and from a different ant (08031), as well as for different laser pulse energy (10 pJ, 20 pJ, 100 pJ for 07911, 08031, and 07910 respectively). The count values for the three ant specimens have been multiplied by ten to offset them from the standards for easier visibility. The mass-to-charge ratios for the identified zinc-containing molecular fragments are indicated by labeled vertical lines in the three stacked “zoomed” plots (only one of the ant spectra is shown in zoomed plots). The peaks for zinc-containing fragments are offset to lower ratios (lighter and faster) from the peaks for fragments containing the same number of nucleons but consisting of only C, H, O and N, because zinc’s higher nuclear binding energy results in less mass-energy per nucleon^87^. This slight “leftward” offset (especially visible as an offset from 117 in the bottom “zoomed-in” plot of the ZnN_2_C_2_H^+1^ spectral region), was found to be useful in discriminating zinc-containing fragments from the many organic fragments, as was the pattern of the 1:0.56:0.38 natural abundance ratios of the ^64^Zn, ^66^Zn and ^68^Zn isotopes. The ant teeth and the two standards all appear to produce Zn^+1^ and ZnNC^+1^ fragments, although, for zinc picolinate, the wide “tails” to the right of the ZnNC^+1^ peaks suggest that a large fraction of the fragments arrive late, for unknown reasons. The Zn^+1^ and ZnNC^+1^ fragments observed from the two standards are consistent with original binding: Zn is bound to N which is, in turn, bound to C in both standards^88,89^. (**B**) Reconstruction of the origins of all zinc-containing molecular fragments (one red dot each) from the FIB-SEM milled, needle-shaped specimen of a distal tooth. The distribution of zinc appears to be homogeneous on the scale of the resolution of the reconstruction and tests did not reveal clustering. But, because of the homogeneity, a different image is needed to place a limit on the resolution. (**C**) APT reconstruction of cuticle from an ant leg with a fragment interface, used to establish an upper limit to the resolution of the technique on arthropod cuticle. The abruptness of the interface between C^+1^ and CO_2_^+1^ fragments, visible in the reconstruction, is quantified in the “Proxigram” which suggests that the resolution of the reconstruction is 7 nm or smaller. The nature of this interface is not known, but a similar interface was found in two other specimen needles from the same leg region.

HEBs appear to be homogenous on previously examined scales (see “Introduction”). We examined the Zn-HEB on a smaller scale using APT. Figure 6B shows that there was no evidence of regions with different composition in the reconstructed origin of molecular fragments that contained zinc-atoms from the Zn-HEB of an ant (*Atta cephalotes*) mandibular tooth. There was also no evidence of clustering in three other similar samples. Figure 6C shows structure in ant leg cuticle that suggests that the resolution of the technique for these specimens was 7 nm or less, and was included because the Zn-HEB was too homogeneous to demonstrate resolution. Structure in several reports of APT of biological materials suggests that the resolution can be 5 nm or less^61,63,64,66,68^. The homogeneity of our results therefore suggests that any zinc clustering within the ant tooth Zn-HEB is on a scale of a few nanometers or less.

The molecular fragments observed from APT (Fig. 6A) further suggest that the ant mandibular tooth material may be homogenous at an even smaller scale, with much of the detected zinc bound as isolated atoms as ZnNC^+1^ (53%) and ZnN_2_C_2_H^+1^ (14%) while the remaining 33% were Zn^+1^. Previous XAS data preclude zinc binding to carbon^13^, so most of the zinc is apparently bound to nitrogen. This is consistent with the hypothesis that the zinc is mainly bound as individual atoms to nitrogen in an organic matrix, such as to nitrogen in imidazole rings of histidine residues in proteins. However, as mentioned in the introduction, the quantity of zinc in ant mandibular teeth is too high for each atom to be bound to four histidines in the typical zinc binding motif^75,90^. Instead, it may be that the dominant binding motif is to a single histidine and water. In other HEBs, with higher concentrations of zinc than in the ants, such as scorpions (see Fig. 4G–J and caption), there may be too much zinc even for binding to a single histidine, and we speculate that some of the zinc may be present in highly amorphous zinc hydroxides.

The argument that the APT fragments indicate binding of individual zinc atoms to nitrogen in the organic matrix, depends on the detected fragments being representative of the original structure rather than products of recombination events. To investigate fragments from zinc-organic binding, we examined standards with Zn bound to N and O: glycine zinc salt monohydrate (C_4_H_10_N_2_O_5_Zn) and zinc picolinate (Zn(C_6_H_4_O_2_N)_2_. We did not find molecular fragments that were inconsistent with original fragments of these molecules (whether the fragments contained Zn or not), and these standards produced ZnNC^+1^ and Zn^+1^ fragments like the ant HEB (Fig. 6A). In the standards, Zn is bound to O as well as to N, but we found no compelling evidence of fragments containing both Zn and O. Thus, the lack of identified Zn- and O-containing fragments from the ants does not preclude binding of Zn to O in the Zn-HEB.

We used ToF–SIMS to confirm the APT fragment identification (the ToF–SIMS instrument had better mass-to-charge resolution), and to look for similar fragments in other species (sample preparation was easier than APT needle preparation). We examined Zn-HEB regions of leafcutter ant (*Atta cephalotes*) mandibular teeth, spider (*Araneus diadematus*) fangs, and nereid worm (*Neanthes brandti*) jaws and found fragments similar to those from the ant APT study, namely, Zn, ZnNC, and ZnN_2_C_2_ in Zn-HEB regions of each of these organisms. We also found other Zn-containing fragments, but we are less confident that the ToF–SIMS fragments represent the original structure than for the APT fragments, because we did not investigate fragments from standards, and because the maximum energy density (and hence the possibility of recombination) was greater in our ToF–SIMS than in our APT studies. The ToF–SIMS fragments were consistent with the model of Zn binding to histidine imidazole nitrogen and oxygen from water.

## Discussion

### Property charts and correlations

Figure 5 shows property charts of related pairs of mechanical properties for the main species, along with comparison species, engineering materials, biomineralized materials, and bromine-HEBs.

Figure 5A shows a strong correlation between hardness and modulus of elasticity for the tested materials. This correlation means that the materials that deform less under pressure in an immediately recoverable manner (higher modulus of elasticity), also tend to suffer smaller lasting indentations (higher hardness).

The HEBs in Fig. 5A tend to deviate from the fit line towards higher hardness, in contrast to the calcified materials, which deviate more towards higher modulus of elasticity. While the crab claw and salmon tooth have a higher modulus of elasticity than the HEBs, they are similar in hardness.

Figure 5B,C show the results of our measurements for pairs of desirable properties that tend to be anti-correlated rather than correlated, resulting in “trade-offs”. The first trend is the tendency for materials with a higher modulus of elasticity to require less energy to fracture^85,91^. The second trend is for materials with higher modulus of elasticity (and hardness, considering the correlation) to be less damped^76^ (this trend is not particularly evident for the materials in Fig. 5C).

Both the damping and the fracture trade-offs with modulus of elasticity can be explained by similar mechanisms. Damping tends to be greater for lower modulus of elasticity materials because, for a given energy used in deforming the material, the deformation is greater, resulting in more relative displacement within the material and more energy lost in internal friction and re-arranging the material. Similarly, energy spent on greater internal re-arrangement in lower elastic modulus materials adds to the energy cost of fracture.

#### Hardness and modulus of elasticity vs. energy of fracture

Two of the tested Zn-HEB non-HEB pairs, the scorpion sting and the ant mandible, followed the general trend towards lesser energy to fracture with higher elastic modulus. And two, the worm and the spider HEBs, had about the same energy of fracture notwithstanding their higher modulus of elasticity.

The ant and scorpion Zn-HEBs, which required significantly less energy to fracture than their paired non-HEBs, had, nevertheless, similar values of a force-challenge^92^ figure of merit. This is because susceptibility to fracture depends on the energy available for fracture as well as the energy required per unit area of fracture. The stored energy is equal to the force multiplied by the incremental distance that the material compresses (for equal forces, more energy is stored in the more compliant spring). Thus, for a given force and shape, less energy is stored in a material that has a high modulus of elasticity. The downward sloping lines in Fig. 5B connect regions with the same values of this force-challenge figure of merit. The ant and scorpion Zn-HEBs and non-HEBs nearly lie along a constant-susceptibility line, suggesting that the HEBs are only slightly less resistant to fracture for a force challenge than their paired non-HEBs.

Figure 5D shows hardness instead of modulus of elasticity plotted against energy of fracture. In this plot, the downward sloping lines are for an analogous figure of merit: they connect regions with the same value of the product of hardness and energy of fracture.

#### Hardness and modulus of elasticity vs. damping

The product of modulus of elasticity and loss tangent has been used as a figure of merit for material damping^76,91^. The average of this figure of merit for the 6 HEBs tested here is significantly greater than the average for corresponding non-HEBs (p = 0.02). The product of the hardness and loss tangent (see Fig. 5C) is also greater on average for the tested HEBs (p = 0.01). This suggests that the HEBs are well damped for their hardness and modulus of elasticity relative to adjacent non-HEB regions.

### Comparison of Zn- and Mn-HEBs to engineering materials and biomineralization

In order to accurately compare HEBs to other materials, we tested a variety of materials using the same techniques that were used for the HEBs. Results for these other materials are also shown in Table 1 and Figure 5.

#### Hardness

Figure 5A shows that the Zn- and Mn-HEBs were at least a factor of 2 harder than the hardest of the tested plastics (PMMA), with a hardness slightly greater than aluminum, but about 1/3 that of the 430 stainless steel and 1/10 of that of the fused silica.

The Zn- and Mn-HEBs were nearly as hard as the calcified materials in crab claws and salmon teeth but only 1/5 as hard as the Fe-biomineral in the chiton teeth. Thus the permanent deformations, pock marks, and scratches in HEB—teeth would be about the same as if the teeth were made of a calcium biomineral or aluminum.

#### Modulus of elasticity

The Zn- and Mn-HEBs were about a factor of 2 stiffer than the stiffest plastics and 1/10th as stiff as fused silica. While the Aluminum was about as hard as the HEBs, it had a significantly greater modulus of elasticity.

The Ca-biominerals were significantly stiffer than the HEBs, though they also were similar in hardness. The HEBs were about about 1/5 as stiff as the magnetite in chiton teeth.

#### Energy of fracture

The HEBs required orders of magnitude more energy to fracture than glass, and the Zn-HEBs required several times more energy to fracture than acrylic.

The HEBs were similar in range to the Ca-biominerals. They required over an order of magnitude more energy to fracture than the chiton Fe-biomineral. The high hardness and modulus of the Fe-biomineral but low energy of fracture illustrates the trade-off between these properties.

#### Damping

The indentation loss tangent of HEBs, Fig. 5B, was high relative to fused silica, in the same range as the plastics, and lower than the loss tangent of the metals in the plastic regime probed by nano-indentation.

The damping of Zn- and Mn-HEBs was similar to that of the calcium biomaterials, but lower than the damping of the Fe-biomineral in chiton teeth.

Some of the Zn-HEBs, as well as the Br-HEB and the Ca-biomineralized tissue that we tested previously^14^, exceeded the Lake threshold of E*tan(δ) = 0.6 for highly damped engineering materials^76^. However, it is important to point out that our values are based on indentation measurements that produce plastic deformations—the damping values of the metals, for example, are much higher than for measurements in the elastic regime. Indentation damping values are likely to be relevant for indentation-like interactions, such as those between cutting and puncturing “tools” and their targets. Greater damping may reduce the likelihood of fracture initiation for these interactions.

#### Impact resistance

As shown in Table 1 and Fig. 5E, all of the tested materials were more impact resistant than either the fused silica or the polycarbonate, with the worm jaws requiring about seven times more impact energy to fracture than polycarbonate, which is often used for its high impact resistance.

#### Abrasion resistance

Figure 5F and Table 1 show that most of the HEBs were more abrasion resistant than the tested plastic, the non-HEBs and the Ca-biominerals. The chiton Fe-biomineral, like fused silica, was much more abrasion resistant than the HEBs, at the expense of a low energy of fracture. The chiton scrapes algae off of rocks, hence the likely need for high hardness and abrasion resistance. The trade-off is the low energy of fracture, but the scraping interaction is slow and there is likely little energy stored in elastic deformation of rock with a thin layer of algae. In addition, the chiton teeth are continually replaced.

### Differences between mechanical properties of Zn- and Mn-HEBs

In the organisms that have both Zn- and Mn-HEBs, the Zn-HEBs were significantly harder (p < 0.012) and had significantly higher modulus of elasticity (p < 0.03) than the Mn-HEB in the same organism. And, as mentioned above, the Mn-HEBs were significantly harder and had a significantly higher modulus of elasticity than any of the tested non-HEBs. This result may explain the observation that Mn-HEBs are often located between the plain- and zinc-materials (see the similar motif in scorpion stings and worm jaws of Fig. 1G). Smaller steps in mechanical properties would reduce the tendency to concentrate stresses that may lead to fracture at interfaces between materials^93^.

We did not find consistent significant differences between all Zn- and Mn-HEBs in abrasion resistance, loss tangent or impact resistance. For example, the Mn-HEB material in the spider marginal teeth was significantly more abrasion resistant in our test than the fang (p < 0.004) but the Mn-HEB in the shaft of the scorpion sting was not significantly different than the Zn-HEB tip.

### Comparison of HEB regions to analogous but non-HEB regions in other organisms

Two of our comparison organisms, a tarantula and a leaf cutter bee were selected because they had non-HEB regions that were analogous to Zn-HEB regions in our model organisms. The Zn-HEB in fangs of the tarantula *Aphonopelma hentzi*, is more localized at the tips than in most other spiders^7^. The hardness and modulus of elasticity of the non-HEB region of the shaft of the tarantula fang was similar to the non-HEB region, rather than to the analogous Zn-HEB region of the shaft of the *A. diadematus* fang (Table 1 and Fig. 5A)*.* Leaf cutter bees, *Megachile rotundata,* which cut leaves for nest building but not energy harvesting, are, like ants, Hymenoptera, but, like other examined bees, do not have Zn-HEBs in the mandibular teeth^7^. The distal regions of the bee mandibular teeth were hard for non-HEBs, but not quite as hard as the least-hard (the nereid worm jaw) of the Zn-HEBs (Table 1 and Fig. 5A,C). These results add further evidence to the argument that HEBs are necessary to produce the highest values of hardness and modulus of elasticity in the cuticle of these organisms.

### Homogeneity scale difference between HEBs and biominerals

In contrast with the HEB homogeneity scale of a few nanometers or less (Fig. 6), the granularity of biomineralized structural tissues tend to be thousands of times larger. The smaller scale homogeneity of the HEBs may be important for very sharp or precision “tools”.

**Figure 7:**
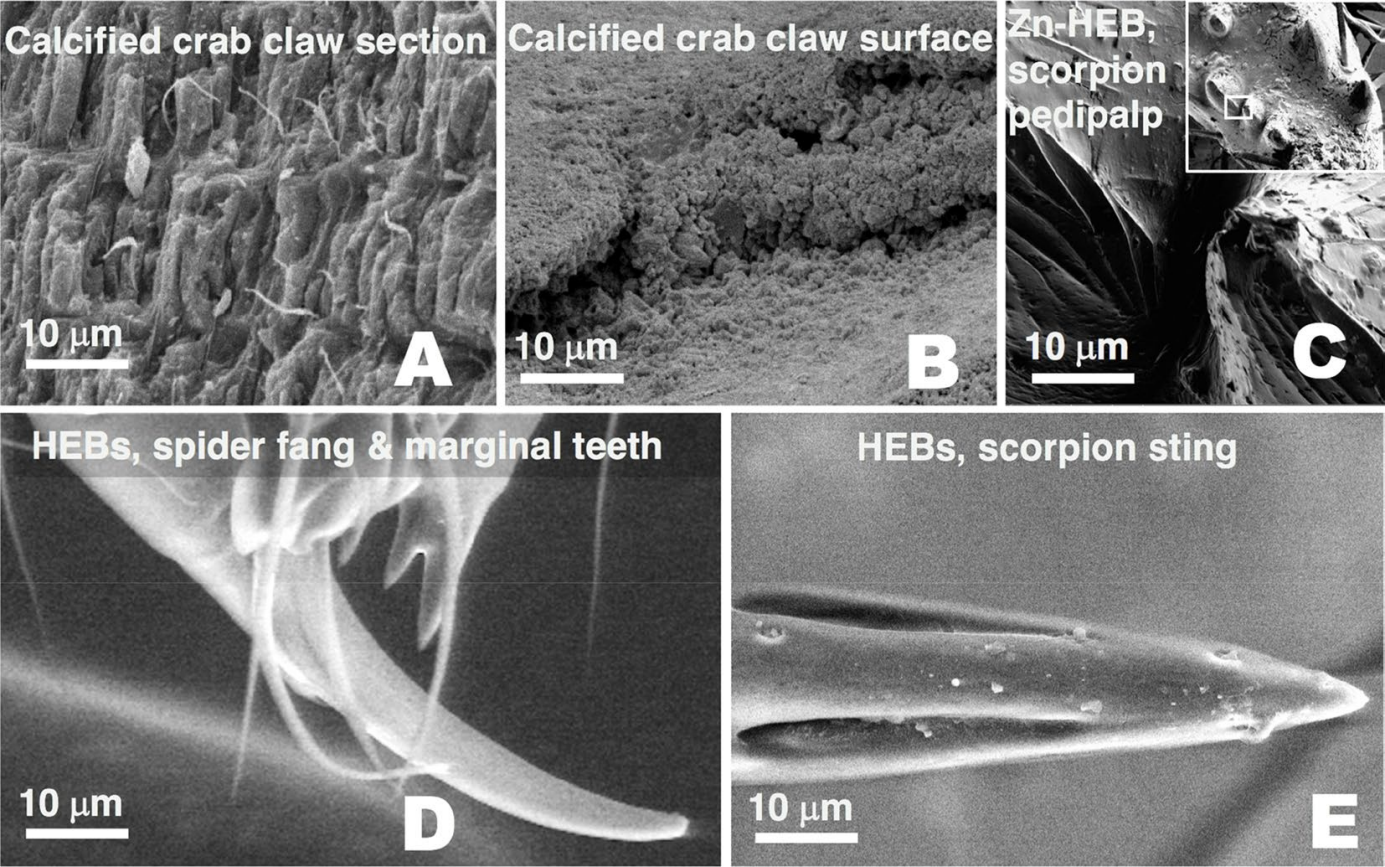
Biomineral inclusions in a crab claw are large compared to structure in HEB-containing “tools” from other arthropods. For comparison, all images are at the same magnification. (**A**, **B**) Calcified regions of a cheliped (large claw) of the crab *Pachygrapsus crassipes*; showing a fracture plane perpendicular to the surface (**A**) and a naturally worn surface (**B**). The calcite inclusions are about 2 × 2 × 15 μm in size and are arranged with their long axis perpendicular to the cuticle surface. (**C**) Portions of two teeth on the pedipalp of a scorpion, *Centruroides exilicauda.* The region is shown in the small white rectangle in the inset image of the pedipalp tip. (**D**) Fang and smaller marginal teeth of a first instar of the common house spider *Parasteatoda tepidariorum.* (**E**) Sting tip from an early instar of the scorpion *Vaejovis spinigerus*. Many structural features are smaller than a single biomineral inclusion in the calcified cuticle.

The biomineral inclusions in crab cuticle are typically several microns in scale (Fig. 7A,B), as are magnetite inclusions in chiton teeth^94^. Human enamel rods are about 5 microns in diameter, though composed of individual crystals that are tens of nanometers in width and thickness^95,96^. The scale of these biomineral inclusions is too large to produce features like those in the HEB structures in Fig. 7C–E. The sharp marginal tooth of a small spider in Fig. 7D is about the size of a single biomineral inclusion in the crab cuticle, Fig. 7A.

Even the smallest reported biomineral crystals would not produce features that are smooth on the nanometer-scales of the cutting edge of leafcutter ant mandibles and ridges on scorpion pedipalp teeth (Fig. 1E,F) and a tip or edge that was the diameter of single crystals would be especially susceptible to fracture at the crystal interfaces. Individual bone crystallites are reported to be the smallest biogenic crystals known^97^. They are only 2–6 nm thick, but 30–50 nm wide and 60–100 nm long. Magnetite crystals in magnetotactic bacteria and human brains tend to be 50 nm in size^98,99^, although crystals can be produced down to 10 nm in scale^100^. The nanoparticles that make up diatom skeletons are also roughly 50 nm in size^101–103^.

### The biological advantages of HEBs: reduction in force, energy and muscle mass

Our results suggest that the homogeneous HEBs enable sharper, more precise “tools” than biomineralization, and better resistance to deformation and damage than plain materials. But are these differences large enough to matter? Here we attempt to translate differences in material properties into biologically relevant energy and muscle mass advantages by comparing the forces required to employ “tools” that utilize the different materials. As a first step, we estimate forces using simple analytical models, rather than numerical models of specific “tools” ^28,104–106^ and targets. This allows us to estimate the scale, and thus the importance, of the force reductions associated with HEBs in general, using Hertzian contact mechanics and empirical models that include inelasticity and abrasion.

#### HEBs vs. biomineralization

First, we illustrate the force reduction possible with smaller diameter tips, comparing the diameter of small HEB tips to the smallest reported biomineralized tips. We noted above that individual mineral inclusions in crab cuticle, chiton radular teeth, and tooth enamel, are on the scale of microns. This scale is consistent with the sharpest reported biomineralized vertebrate teeth, the teeth of extinct conodonts^107^ with 2 μm diameter tips (in contrast with pristine baby mouse teeth, 364 μm^23^, and microchiropteran (bat) molar tips, 25 μm^43^). The tips of the spider marginal teeth (Mn-HEB) at the center of Fig. 7D are about 1/10 the diameter of the tips of the conodont teeth. In a simple model, a contact region that was a factor of ten smaller in diameter would require a factor of 100 lower force in order to reach the pressures required to initiate puncture of thick targets^23^. The lower force can lead to a proportionally lower caloric requirement, because the energy is equal to the integral of force times the incremental distance over which it is applied, and the distance is approximately independent of the force. We have verified that both the force and the required energy expenditure are approximately proportional to contact area or to contact diameter when puncturing a thick or thin target, respectively, with real teeth, stings and claws held in a test apparatus^23^. These results illustrate the great reduction in required energy expenditure and force (and thus muscle mass), that is possible with smaller contact areas in puncturing and cutting “tools”.

#### HEBs vs. plain materials

HEBs can also have smaller contact areas relative to plain organic tissues, not because the “tools” can start off sharper, but because they would tend to be blunted less by elasticity, viscoelasticity, plasticity and abrasion.

##### Elasticity

In order to estimate the advantage conveyed by the higher modulus of elasticity associated with HEBs, we model the tip of a tooth or sting as a sphere, and an edge, such as a blade edge, as a cylinder, interacting with a planar surface of the target, under the Hertzian approximation that both the “tool” and target materials can be modeled as linear elastic materials as the stress develops that leads to cutting or puncture.

The stiffer “tool” tip made of the HEB will deform less than an otherwise identical tip made of the non-HEB as it presses against the target. As a result, the force is applied to a smaller contact area, focusing the force onto a smaller area of the target and thus producing the same pressure using less force. The maximum pressure, *p*_*0*_, is, for the hemispherical tip, given by (for more detail on these equations, see basic contact mechanics references, e.g. Williams and Dwyer-Joyce^108^):

$${p}_{0}=\frac{3F}{2\pi {a}^{2}} ,$$

where “*F*” is the force applied by the animal, and “*a*” is the radius of the contact area between the tip and target, determined by the deformation of each surface, and given by:

$$a={\left(\frac{3FR}{4{E}^{*}}\right)}^\frac{1}{3} ,$$

where “*R*” is the reduced radius of curvature, which, for the case of a sphere on a plane, is simply the radius of the sphere, and “E*” is the reduced modulus of elasticity, defined as:

$$\frac{1}{{E}^{*}}=\frac{\left(1-{\nu }_{tool}^{2}\right)}{{E}_{tool}}+\frac{\left(1-{\nu }_{target}^{2}\right)}{{E}_{target}} ,$$

where “*v*” refers to the Poisson’s ratio for the materials, and the subscripts “tool” and “target” refer to the contacting regions of the tool and its target material.

Assuming that the same maximum pressure is required to puncture the same target, the ratio of the force for the spherical HEB tip, *F*_*HEB*_, to the force for the non-HEB tip with the same radius*, F*_*nonHEB*_, can be derived from the above equations and is:

$$\frac{{F}_{HEB}}{{F}_{nonHEB}}={\left(\frac{{E}_{nonHEB}^{*}}{{E}_{HEB}^{*}}\right)}^{2}(spherical\;tip),$$

where “E*_nonHEB_” is the reduced modulus, given above, for the non-HEB tip—target interaction, and “E*_HEB_” is the reduced modulus for the HEB tip interacting with the same target. Similarly, for an edge instead of a tip, modeled as a cylinder interacting with a plane, we have:

$$\frac{{F}_{HEB}}{{F}_{nonHEB}}=\frac{{E}_{nonHEB}^{*}}{{E}_{HEB}^{*}} \left(cylindrical\;blade\;edge\right).$$

The force advantage given by the HEB over the non-HEB is greater for targets with higher moduli of elasticity. For example, for a leafcutter ant using the tip of its distal tooth (E = 9.2 GPa, *v* assumed to be 0.4) to puncture the non-HEB mandibular cuticle of an attacking ant (E = 5.3 GPa, *v* assumed to be 0.4), only 0.62 as much force would be required than if the puncturing tooth were made of the non-HEB mandible material (E = 5.3 GPa).

In contrast, the advantage is much less (0.98 vs. 0.62) for puncturing a leaf which has a much lower modulus of elasticity (E = 0.1 Gpa^109^). This is because the contact area is largely determined by deformation of the low-modulus leaf.

Thus, the main advantage of the higher modulus of elasticity associated with HEB-tipped tools is apparently not in cutting leaves, but in puncturing and cutting stiffer materials such as those that might be encountered in trail maintenance or in defense against army ants or other arthropods. An ant employing HEBs could potentially produce the same puncture or cutting damage to an attacker with 0.62 of the energy required for an ant without HEBs, and, equally or more important, 0.62 of the required mandibular muscle mass (assuming that the maximum force is proportional to the cross sectional area of the muscle and the muscles are the same length and density in each case). The mandibular muscles are the largest muscles in ants^110^, and a reduction in required muscle mass would reduce the energy cost to the colony of producing and maintaining an ant that could defend effectively against that attack.

For the scorpion, the case is similar: the Zn-HEB at the tip would reduce the necessary force by a factor of about 0.6, relative to the non-HEB, when puncturing a sclerite with the elastic modulus of the ant non-HEB (E_Zn-HEB_ = 12 GPa, E_nonHEB_ = 6 Gpa, E_target_ = 5.3 GPa), but virtually no difference in puncturing resilin (0.002 GPa^111^) located between sclerites. The higher modulus of elasticity of the HEBs would give the spider and nereid worm similar advantages.

In addition to lesser deformation of the contact region at the tip or edge of the tool, the HEB’s higher modulus of elasticity would also reduce bending of the full HEB structure. A tooth with twice the modulus of elasticity will tend to deflect half as much under the same lateral force. HEBs may thus reduce the need for careful “tool” alignment for minimizing lateral forces.

A potential drawback of a higher modulus of elasticity is that it tends to be correlated with a lower energy of fracture, and a fracture could increase contact area and obviate the advantages discussed here. However, as mentioned in the “Property charts and correlations” section, two Zn-HEBs actually had significantly lower susceptibilities to fracture in a force challenge than the non-HEBs, according to the figure of merit. The two Zn-HEBs with the slightly higher fracture susceptibilities, from the ant and scorpion, had significantly higher energy damping than the non-HEBs, which likely reduces fracture susceptibility and is not accounted for in the quasi-static figure of merit.

##### Plasticity and viscoelasticity

The tip or blade blunting associated with elasticity is compounded by blunting associated with plasticity and viscoelasticity (which differ from elasticity in that the tip shape never recovers or takes extra time to recover, respectively). We estimate the ratio of elastic to inelastic deformation in tips and edges by assuming that it is the same as for our AFM indentation measurements (we do not compare the hardness because it is not an exclusive measure of plasticity). We estimate the ratio of forces for the different materials using the approximation that the change in shape of the tip or blade, as it is forced into the target, is the same as it would be for elastic blunting, but with a lower “effective” modulus of elasticity to account for both elastic and inelastic deformation. Comparison of insertion and withdrawal portions of the force–displacement curves from the AFM indentations indicated that the ratio of inelastic to elastic deformation was large, about 2.2 for the Zn-HEB in the ant teeth, and 2.6 for non-HEB ant cuticle. While the inelastic effects substantially increase the required force for both types of materials, the change in the ratio of forces, HEB vs. non-HEB, is relatively small, 0.57 instead of the 0.62 calculated above assuming elasticity only.

It is important to emphasize that plastic blunting is more deleterious than similar elastic or viscoelastic blunting because it is permanent. A single high-pressure encounter would make subsequent lighter tasks, that would not have deformed the tip as much, cost extra energy, possibly for the remaining life of the organism. We estimate that the minimum diameter of the ant’s Zn-HEB tooth tip, after puncturing ant cuticle, would be roughly 0.8 μm (compared to the 0.1 μm diameter for pristine mandibles^38^), based on the volume of material plastically displaced in indentations that produced pressures similar to those expected to puncture ant cuticle. One might predict that younger leaf-cutter ants would avoid higher-pressure tasks, like defense against arthropods and cutting of sticks that fall on trails, leaving them to older adults for whom the integrated energy cost would likely be lower.

##### Abrasion resistance

Lower force and muscle mass requirements are also likely to be the primary advantage of abrasion resistance. At first, the HEB tip and an otherwise identical non-HEB tip would require the same force to initiate puncture (we conservatively assume identical deformation in order to separate out the effect of abrasion resistance). But the tip of the tool with the lower abrasion resistance would abrade away more, causing a greater increase in its diameter, and the higher force required for the newly wider tip would increase the abrasion rate, further increasing the diameter and the required force. Because of this feedback, the required force can become strongly dependent on abrasion resistance. For a simple model, the incremental volume lost, “ΔV”, is given by ΔV = F/B Δx, where “Δx” is the effective incremental distance over which the force, “F”, is applied and “B” is the abrasion resistance (Joules/cubic meter). The model is based on three assumptions: first, that “B” is independent of the force, which we confirmed over a factor of ten in force for crab cuticle^14^, second, that the puncture force for thick targets scales as the square of the tip diameter, F *α *d^2^, which we have demonstrated with biological “tools”^23^, and third, that the tip is conical, with the diameter proportional to the distance from the apex, a reasonable approximation for most “tools” in Figs. 1, 4 and 7. The solution suggests that abrasion causes the required force to increase as the square of the total effective distance traveled in or on the abrading material. An interesting outcome of the model is that, even as the forces increase, the ratios of the forces required for tips with different abrasion resistances is constant, once the diameters of the abraded tips are large compared to their original diameter:

$$\frac{{F}_{HEB}}{{F}_{nonHEB}}\approx {\left(\frac{{B}_{nonHEB}}{{B}_{HEB}}\right)}^{2}\left(\mathrm{for\;a\;thick\;target\;and\;tip\;diameters\;determined\;by\;abrasive\;wear}\right),$$

where the abrasion resistance, “B”, can be approximated by the values in Table 1 from our abrasion test. For the scorpion sting, this model suggests that the greater abrasion resistance of the Zn-HEB provides a great advantage, requiring only 0.22 as much force to initiate puncture on a given target once the tip diameter is entirely determined by abrasion. For the nereid worm, which is surrounded by abrasive sand in nature, only about 0.16 as much force would be required to puncture targets with the Zn-HEB instead of the non-HEB, after significant abrasion of the jaw tip.

These estimates demonstrate that HEBs can significantly reduce requirements for force, muscle mass and energy for essential activities. More generally, these results suggest that even minor modifications of material properties may have strong adaptive advantages.

## Summary

The main advantage of the zinc- and manganese-containing materials, relative to plain organic structural materials, appears to be that they are stiffer (higher modulus of elasticity) but nevertheless, equally or more damage resistant, as indicated by their high hardness, abrasion resistance, damping, and energy of fracture. Their high hardness indicates that they are especially resistant to permanent plastic deformation that reduces the efficiency of sharp “tools” and mating surfaces (such as in scissors and forceps). The main advantage of the zinc- and manganese-materials, relative to the tested biomineralized materials, is likely their homogeneity, which may enable sharper and more precisely shaped structures since HEBs do not have mineral inclusions. In addition, the homogeneity of HEBs relative to biomineralization, may reduce chipping damage like that found along razor blades at boundaries between grains of differing stiffness^112^.

While HEBs appear to have mechanical property advantages over plain materials, and homogeneity-related advantages over biomineralized material, they do not provide unique ranges of the mechanical properties we measured, although they may provide unique combinations of these properties. If the calcified materials examined here were used in place of the HEBs, the tools could be even stiffer, and, in the bulk, roughly as damage resistant. On the other hand, they could not be nearly as sharp or have as precisely mated surfaces, and would probably not be as damage resistant when the scale of structural features, like edges and tips, approached the micron scales of the mineral inclusions.

The precise edges, tips and other shapes made from HEBs may be possible because they start off as protein structures, taking advantage of the exquisite control developed for proteins. The mechanical properties of the protein structures are then improved late in cuticle development by depositing large numbers (up to about 20% by mass) of individually bound heavy atoms that fill the spaces between the proteins in the already-rigid and sclerotized cuticle.

The APT data, along with the EXAFS and amino acid data discussed in the introduction, suggest that the majority of the zinc is bound as individual atoms to nitrogen atoms, likely of histidine imidazoles. While there is too much zinc for it to all be involved in cross linking histidines, a minor fraction may increase the modulus of elasticity and hardness by cross linking. There are many examples of materials that do not meet our somewhat arbitrary threshold of 1% heavy elements for HEBs, such as insect ovipositors ^113,114^, but which contain sub-percent levels of zinc, bromine or other heavy elements. It is possible that these lower levels of heavy elements affect material properties through the cross-linking mechanism, but this needs to be confirmed with mechanical tests.

The zinc that is not involved in cross links may reduce plasticity by filling intermolecular spaces in the cuticle, increasing hardness by reducing the space for relative motion under high pressure, and by binding water to proteins, reducing lubrication of relative motion of the macromolecules. Heavy elements may also increase damping of high frequency vibrations by lowering resonant frequencies of the binding molecules^14^. Finally, large quantities of zinc bound to histidine and water may act as a reservoir for quickly replacing the smaller quantities of histidine–zinc–histidine cross links when high forces cause the cross links to break. The idea would be that the high availability of zinc would increase the efficiency of a damping and “self-healing” mechanism similar to that proposed for mussel byssus threads^115^.

The multiple hypothesized mechanisms provide a possible evolutionary path for the gradual development of Zn-HEBs that would be advantageous at every step. At first, small quantities of zinc would improve mechanical properties by increasing cross links in the cuticle. These cross-linked materials would eventually evolve into materials with high concentrations of zinc that enabled the hypothesized space-filling, water immobilization and/or damping mechanisms.

The results here tend to further support inclusion of the zinc- and manganese-rich materials, found in distantly related organisms, together under broad zinc- or manganese-HEB classes. The clustering of the materials from very different organisms into the same regions of material property charts, here, add to the previously noted similarities in elemental associations, such as to Ca and Cl^12^, locations in contact surfaces of “tools” of distantly related organisms^12^, similar developmental courses^32^, and similar chemical environments^13^. The detailed similarities across distantly-related organisms is evidence supporting early evolution of HEBs.

Further study of HEBs may reveal new biochemistry and increase understanding of the importance of material properties for organisms and their interactions with their environment. Man-made materials based on HEBs might find application in, for example, sharp medical devices. More generally, we would argue that there are strong pressures for evolution to develop materials that are particularly good at maintaining sharpness and precise shape during cutting and puncture, and so, when these properties are desired, natural materials are good candidates for biomimetic materials.

The ability to produce nanometer-scale hard features and the associated force and energy savings may be part of the reason that HEBs are common. Our estimates suggest that the use of HEBs in force-focusing structures can significantly reduce force and energy requirements through greater sharpness, lesser deformation during usage, and lesser degradation from damage. Our estimates for Zn-HEBs, relative to non-HEBs, suggest that 0.6–0.2 of the force, muscle mass and energy can be required to initiate puncture and cutting, and even more advantageous factors relative to biomineralized materials with typical sizes of mineral inclusions. The smaller forces may make food and energy sources available to small, force-limited organisms, and the energy savings associated with the smaller forces may be important even for larger organisms.

These results highlight the importance of material properties in reducing the forces and energy required by organisms for defense and for obtaining energy. Heavy element biomaterials may make food and energy sources available to small, force-limited organisms, and the energy savings associated with the smaller forces may be important even for larger organisms. HEBs may allow for smaller, less costly offspring by reducing required forces, they may also make longer lives possible by reducing “tool” damage, and they likely play an essential role in the ecology and evolution of a broad range of organisms.
